# Dissecting Context-Specific Effects of ERK5 Signaling in Triple-Negative Breast Cancer

**DOI:** 10.3390/cancers18030376

**Published:** 2026-01-26

**Authors:** Katherine L. Hebert, Sarah B. Knopf, Thomas Cheng, Megan C. Benz, Bridgette M. Collins-Burow, Jorge A. Belgodere, Frank H. Lau, Elizabeth C. Martin, Matthew E. Burow, Van H. Barnes

**Affiliations:** 1 Department of Medicine, Section of Hematology & Medical Oncology, Tulane University School of Medicine, New Orleans, LA 70112, USA; khebert6@tulane.edu (K.L.H.);; 2 Tulane Cancer Center, Tulane University, New Orleans, LA 70112, USA; 3 Department of Biological and Agricultural Engineering, Louisiana State University and Agricultural Center, Baton Rouge, LA 70803, USA; 4 Department of Medicine, Tulane University, New Orleans, LA 70112, USA; 5 Keliomics, Inc., Portland, OR 97239, USA

**Keywords:** macrophysiological model, ERK5, extracellular matrix, epithelial to mesenchymal transition, MAPK

## Abstract

**Background:** Triple-negative breast cancer (TNBC) is an aggressive subtype of cancer with poor clinical outcomes. There is a critical need to identify novel, druggable targets for TNBC to improve therapy response and patient outcomes. Due to their roles in critical processes driving cancer progression, kinases have been a major focus of drug discovery efforts. The role of extracellular signal-regulated kinase 5 (ERK5) in mediating TNBC extracellular matrix (ECM) has previously been described in 2D culture and in vivo. Here, we characterized the impact of ERK5 on breast cancer biology in 2D culture, 3D spheroids, and our 3D breast adipose-macrophysiological system (BA-MaPS). **Methods:** We assessed migration changes in MDA-MB-231 parental and ERK5-knockout (ERK5-ko) cells cultured in the three in vitro models using transwell, scratch, and spheroid pseudo-migration assays. Differential gene expression among these cell lines in the three platforms was assessed by RNA sequencing and pathway analysis. Stromal remodeling of adipocytes and matrix was evaluated by H&E and Masson’s Trichrome. **Results:** Across the in vitro models, ERK5 deletion impaired TNBC cell migration. ERK5-mediated transcriptomic changes included genes associated with epithelial-to-mesenchymal transition (EMT) and migration, with further analysis showing significant alterations in core and associated matrisome. Histological staining corroborated the downregulation of collagen with ERK5 depletion in the BA-MaPS. The NFκB pathway was significantly upregulated only in the ERK5-ko 2D-cultured cells, not in 3D spheroids nor the BA-MaPS model. **Conclusions:** These results indicate a link between ERK5 and TNBC progression through regulation of TME remodeling, EMT, and cell motility. Differences in 2D culture, 3D spheroid, and BA-MaPS underscore the importance of using physiologically relevant models in breast cancer research.

## Simple Summary

Drug discovery research is hindered by a lack of models that mimic physiological conditions in cancer patients. Triple-negative breast cancer (TNBC) is clinically aggressive with high rates of relapse and limited treatment options. Kinases are attractive therapeutic targets due to their roles in cell fate, and irregular kinase activity drives tumor progression. Here, we analyzed a mitogen-activated kinase pathway in traditional and complex in vitro models to determine context-specific effects mediated by kinase expression, with the aim of applying these findings in the clinical setting. Our goal is to identify viable treatment targets to improve both overall and regression-free survival of TNBC patients.

## 1. Introduction

Triple-negative breast cancer (TNBC) is an aggressive breast cancer subtype characterized by a lack of both hormone receptor expression and amplification of the human epidermal growth factor receptor 2 (HER2). The deleterious effects of neoadjuvant chemotherapy (NACT), the standard of care for TNBC patients, lead to postoperative complications and mortality. Rates of acquired resistance to NACTs are high, further limiting treatment options [1,2]. There is a vital need to identify viable therapeutic targets to improve patient outcomes.

The mitogen-activated protein kinase 7 (MAPK7)/extracellular signal-regulated kinase 5 (ERK5) pathway is linked to chemotherapy resistance and has been shown to promote tumorigenesis and epithelial–mesenchymal transition (EMT) in TNBC, suggesting its potential as a therapeutic target for TNBC [3,4]. Findings from our group, corroborated by other researchers, demonstrated that ERK5 drives TNBC tumor growth through regulation of the extracellular matrix (ECM) [5,6,7].

Interactions between TNBC cells and the ECM, including the surrounding adipose tissue, are complex and contribute to cancer progression and drug resistance [8,9]. Because cell-to-tissue interactions cannot be analyzed in 2D culture, 3D models offer a more accurate platform for investigating cancer proliferation, progression, and potential therapies [10,11,12,13]. Recently, research and regulatory efforts have reinvigorated the development of new approach models that better recapitulate clinical tumor behavior and response to treatment, such as microphysiological systems and organ-on-a-chip platforms [14,15,16,17].

The focus of this study was to further characterize the role of ERK5/MAPK7 in mediating TNBC aggressiveness using our breast adipose tissue-based macrophysiological system (BA-MaPS) composed of human breast tissue seeded with TNBC cells anchored to the bottom of a well by two confluent breast adipose stromal cell (brASC) sheets [18]. Using this novel in vitro model, we examine how ERK5 differentially alters the transcriptome, EMT, cellular migration, and ECM compared to effects in 2D and 3D spheroid culture using mesenchymal (Basal B) TNBC cell lines MDA-MB-231 and Hs578T [19]. Further, we highlight TNBC remodeling of stromal cells in BA-MaPS, which cannot be mimicked in standard 2D or 3D culture models.

## 2. Materials and Methods

### 2.1. Cells and Reagents

TNBC cell lines MDA-MB-231 and Hs578T were acquired from American Type Culture Collection (ATCC). ERK5-ko cells were previously generated using a pU6-driven guide strand with dual expression cassettes for Cas9/EGFP approach (Horizon, Cambridge, UK) [6]. Cells were cultured in Dulbecco’s Modified Eagle Medium (Invitrogen, Carlsbad, CA, USA, DMEM; pH 7.4) supplemented with 10% Fetal Bovine Serum (Thermo Scientific, Waltham, MA, USA, FBS), 1% nonessential amino acids, minimal amino acids, sodium pyruvate, antibiotic/anti-myotic, and insulin (complete media) under mycoplasma-free conditions.

Normally discarded breast tissue-derived stem cells and breast adipose tissue were acquired from routine surgeries, including breast reduction and breast reconstruction through IRB oversight at Tulane University (2023-356). De-identified brASCs were extracted from tissue through mild mincing and explant culture. Donors for brASC cell sheets and human breast tissue were matched by BMI and/or race, as well as age, to create BA-MaPS. BA-MaPS models were generated by sandwiching minced human breast tissue between two confluent ASC sheets using a thermoresponsive culture plate, as described by Brown et al. [18], and cultured in MEM Alpha (Thermo Scientific, Waltham, MA, USA, #12571063) supplemented with 10% Fetal Bovine Serum Premium Select (R&D Systems, Minneapolis, MN, USA, #S11550) and 1% antibiotic/anti-myotic (ASC media). BrASCs were used between passages 1 and 5. All cells were grown in incubators (37 °C in humidified 5% CO_2_).

### 2.2. Transfection of Turbo-RFP Labeled Cell Lines

MDA-MB-231 and MDA-MB-231-ERK5-ko cells were plated in 10 cm^2^ dishes (VWR, Radnor, PA, USA) at 100,000 cells/mL in complete media. After 24 h, cells were transfected with 5 μg of Lenti-CMV-RFP-2A-Puro-Blank Lentivirus (ABM, Vancouver, BC, Canada, Catalog Number: LVP691) plasmid in 1 mL Optimem (Thermo Scientific, Waltham, MA, USA, Catalog Number: 11058-021) with 30 μL of attractene (Qiagen Inc., Hilden, Germany, Catalog Number 301007). After 24 h, the media was aspirated and replaced with fresh complete media for selection, containing puromycin at 1 μg/mL. After 48 h, cells were transferred to a T75 (VWR, Radnor, PA, USA) with complete media and grown to ~80% confluency. Cells were prepped for Fluorescent-Activated Cell Sorting (FACS) by resuspending 1 million cells/mL in 500 μL of Optimem. The top 5% cells with the brightest RFP signal were sorted on a BD FACSAria Fusion (BD Biosciences, Milpita, CA, USA).

### 2.3. RNA Sequencing and Pathway Analysis

MDA-MB-231, Hs578T, and the respective ERK5-ko cell lines were used for all experiments. Two million cells of each cell line were seeded in an AggreWell 400 24-well plate (Stem Cell Technologies, Vancouver, BC, Canada) that was prepped with anti-adherence solution (Stem Cell Technologies, Vancouver, BC, Canada) in accordance with the manufacturer’s protocol and grown overnight to form 1200 3D spheroids containing about 1700 cells. After 24 h, spheroids were pelleted for RNA extraction. All cell lines grown in 2D culture and 3D spheroid culture were extracted for total RNA using Quick RNA Miniprep Kit (Zymo Research, Irvine, CA, USA) according to the manufacturer’s protocol. RNA was isolated from the adipose tissue-based system using QIAzol Lysis Reagent (Qiagen, Venlo, Netherlands) and Quick RNA Mini-Prep Kit (Zymo Research, Irvine, CA, USA) in accordance with the manufacturer’s protocol. RNA samples were shipped on dry ice to Genewiz (Leipzig, Germany) for RNA sequencing. The RNA sequencing workflow after RNA isolation included initial PolyA selection. Adaptor ligation, polymerase chain reaction (PCR), and Illumina NovaSeq technology-based sequencing with 2 × 150-base pair (bp) read length were carried out. Reads were aligned to GSEA against nine different molecular signature databases using Spliced Transcripts Alignment to a Reference (STAR). The statistical results were generated using DESeq2. Log2FC was used to estimate changes in gene expression, representing the ratio of gene expression between the two conditions. Raw data files are available in GEO (GEO Submission (GSE316523)).

Analysis was performed as follows: data sets for comparison were total gene changes between MDA-MB-231-ERK5-ko vs. MDA-MB-231 parental. All genes were considered significant if the adjusted *p*-value < 0.05. Identification of significantly altered pathways was performed through the Enrichr gene set analysis web server [20,21], specifically from the KEGG database accessed in June 2025.

### 2.4. Histology

BA-MaPS^Parental^ and BA-MaPS^ERK5-ko^ samples were fixed after 5 days in culture in 10% formalin (Sigma-Aldrich, St. Louis, MO, USA), paraffin-embedded, sectioned at 5 μm, and stained with H&E and Masson’s Trichrome by the Histology Core at Tulane University School of Medicine. Slides were scanned with a PhenoCycler^®^ Fusion (Akoya Biosciences, Marlborough, MA, USA) and evaluated for cell structures and ECM fiber content. Visualization of cells and collagen fiber content was through the Inform Tissue Analysis Software version 3.0 (Akoya Biosciences, Marlborough, MA, USA). Experiments were completed in 8 distinct adipose tissue donors (*n* = 8) (Supplemental Table S1). Adipocyte size was quantified through QuPath via the Random Trees (Rtrees) classifier [22].

### 2.5. Western Blot

Cells were grown in 2D culture until ~80% confluence, collected in PBS, and pelleted at 1500 rpm. Spheres were formed in 3D by seeding 2 million cells in an AggreWell 400 according to the manufacturer’s protocol, accounting for roughly 17,000 cells/spheroid. After 24 h, spheroids were collected and pelleted at 1500 rpm. Both 2D and 3D cell pellets were lysed with 100–150 µL of mammalian protein extraction reagent (MPER) supplemented with 1% protease inhibitor and 1% phosphatase inhibitor (Thermo Scientific, Waltham, MA, USA). For the BA-MaPS experiments, half a million MDA-MB-231 parental and ERK5-ko cells were seeded in breast adipose tissue, cultured for 5 days, and then lysed with 500 μL of RIPA buffer supplemented with protease and phosphatase inhibitors. Samples were centrifuged at 15,000 RPM for 15 min at 4 °C; the tissue layer was removed, 1% Triton X-100 was added to the supernatant, mixed well, and kept on ice for 60 min. The mixture was centrifuged again at 15,000 RPM to remove residual lipids. Protein concentration was determined using the PierceTM BCA Protein Assay Kit (Thermo Scientific, Waltham, MA, USA). For all three models, proteins were heat-denatured at 70 °C, and max protein was loaded per lane on NuPAGE 4–12% Bis-Tris Gel (Thermo Scientific, Waltham, MA, USA). Protein was then transferred to nitrocellulose membranes using the iBlot 3 Western blot transfer system per manufacturer’s instructions (Thermo Scientific, Waltham, MA, USA). Membranes were incubated in InterceptTM Blocking Buffer (LiCor Biosciences, Lincoln, NE, USA) for 1 h followed by 4 °C incubation overnight with primary antibodies diluted 1:1000 for loading control and 1:500 for other target antibodies (B-Actin: Cell Signaling Technology, Catalog Number 3700S; NFкB p65/RelA: ABclonal Technology, Catalog Number A10609; phospho-NFкB p65: Cell Signaling Technology, Catalog Number 3033S; NFкB2 p100/p52: Cell Signaling Technology, Catalog Number 3017T; IkBα: Cell Signaling Technology, Catalog Number 4814T; and ERK5: Cell Signaling Technology, Catalog Number 3552S). After three 5 min washes in 1% Tris-Buffered Saline with Tween 20 (TBS-T), membranes were incubated with appropriate secondary antibodies for 1 h, then IR-tagged secondary antibodies (IRDye 800CW, Catalog Number 926-32210 and IRDye 680RD, Catalog Number 926-68072) (LiCor Biosciences, Lincoln, NE, USA) were used at a 1:10,000 dilution in InterceptTM Blocking Buffer. Following incubation with secondary antibodies, membranes were washed three times for 5 min in 1% TBS-T, and blots were analyzed by the ChemiDoc MP (BioRad, Hercules, CA, USA). Band density was quantified through ImageJ version 1.53. Data were normalized to ß-Actin, as a loading control. Experiments were conducted in biological triplicate; BA-MaPS was constructed from 3 distinct breast tissue donors (Supplemental Table S1).

### 2.6. Transwell Migration Assay

MDA-MB-231-ERK5-ko, Hs578T-ERK5-ko, and parental control cells were seeded at 25,000 cells in 300 μL of DMEM with 1% FBS and in an 8.0 μm porous transwell, 24-well format (Corning, Corning, NY, USA). In total, 500 µL of DMEM containing 10% FBS, the chemoattractant, was added to the lower wells. After 26 h, inner membranes were scrubbed to remove non-migrated cells. Cells on the outer membranes were fixed in formalin and stained with 1% crystal violet and 10% methanol solution. The entire outer membrane was imaged, and cells were manually counted. The data represent three independent biological experiments.

### 2.7. Three-Dimensional Spheroid Pseudo-Migration Assay

MDA-MB-231-ERK5-ko, Hs578T-ERK5-ko, and the parental cell lines were seeded at 5000 cells per well in a 96 U-shaped bottom microplate (Thermo Scientific, Waltham, MA, USA) that was non-treated. After 24 h, spheroids were transferred into the center of a 48-well cell culture plate (VWR, Radnor, PA, USA). Images were taken at Day 0, 24 h, 48 h, and 72 h on the BioTek Cytation 5 (Agilent Technologies, Santa Clara, CA, USA). The area around the spheroid was measured through Fiji/ImageJ version 1.53 to determine the migration of the cells over time. The average area of the spheroid was measured over *n* = 3 experiments and normalized to day 0.

### 2.8. Time-Lapse Imaging for Migration Analysis

MDA-MB-231-ERK5-ko and parental cells were seeded in 2D culture or the 3D BA-MaPS, 1,000,000 per system, composed of primary human breast tissue in triplicate (Supplemental Table S1) and assembled in a 100 mm2 Petri dish (VWR, Radnor, PA, USA) [18]. After 24 h, the media was changed, and individual BA-MaPS systems were secured in a stage top incubator attached to a Nikon Eclipse Ti-S. Time-lapse imaging was used to record cell movement and activity using NIS Elements software version 6.10.01. Each video was taken over the course of 6 h and utilized both TRITC (Red Fluorescent Protein) and brightfield imaging, with images being captured every 10 min at 20× magnification. This generated 36 frames for each video, and subsequent video files were exported at 5 frames per second (overall length of video 7.2 s). Cell tracking analysis was completed on the ImageJ version 1.53 add-ons MTrack and ADAPT.

Hs578T parental and ERK5-ko cells BA-MaPS systems were secured in a stage top incubator attached to a Nikon Eclipse Ti. Time-lapse imaging was used to record cell movement and activity at selected points from each treatment (well) using SlideBook ver. 2023.3 (43289) software. Each video was taken over the course of 48 h and utilized both CY3 (Red Fluorescent Protein) and brightfield imaging, with images being captured every 30 min at 10× magnification. This generated 96 frames for each video, and subsequent video files were exported at a rate of around 12 frames per second (overall length of video 7.7 s). Average speed and total displacement were analyzed within the SlideBook particle tracking analysis software version 2023.3 (43289), in which the center of intensity of the CY3 channel for each individual cell was tracked using the Ridler-Calvard thresholding method.

### 2.9. Scratch Assay

MDA-MB-231-ERK5-ko and parental cell lines were seeded on a 6-well 2D culture plate (VWR, Radnor, PA, USA) and grown to ~90% confluency. A wound or “scratch” was made in the center of the well by taking a 10 µL pipette tip and drawing a line down the center of the well. Time-lapse images were taken every 30 min over 24 h on a HoloMonitor Live Cell Imager (Phase Holographic Imaging PHI Inc., Boston, MA, USA) and analyzed using the wound healing assay on the HoloMonitor App Suite cell imaging software version 4.0.0.535 (Phase Holographic Imaging PHI Inc., Boston, MA, USA).

### 2.10. Statistical Analysis

Statistical analyses were performed using Graphpad Prism software version 8.4.3. Data were subjected to an unpaired Student’s t-test. Studies involving more than two groups were analyzed by one-way analysis of variance (ANOVA) or two-way analysis of variance followed by Tukey’s post hoc multiple comparison tests.

## 3. Results

### 3.1. ERK5-Mediated Gene Expression Differs Across Culture Systems

**Figure 1:**
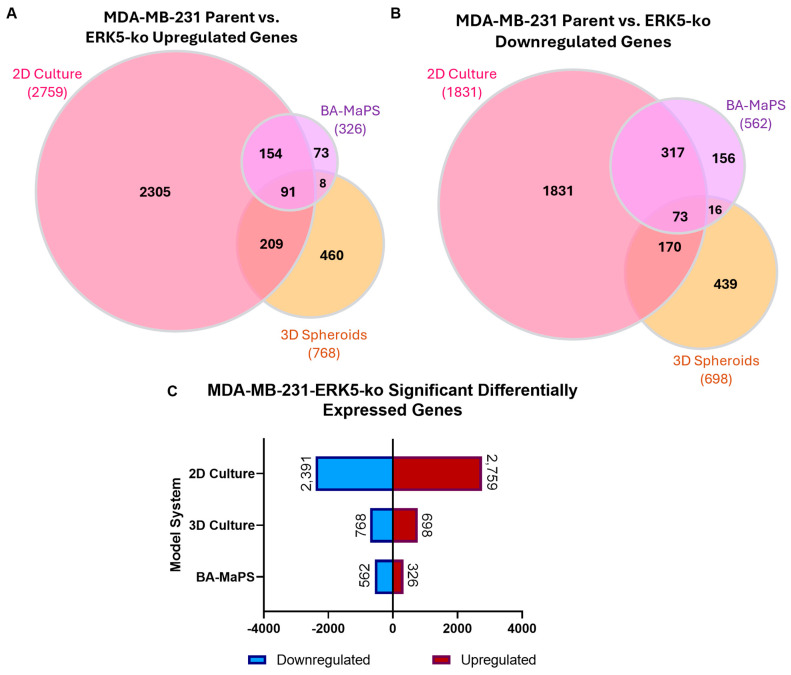
Progression of 3D model systems exhibits reduced transcriptomic alterations following ERK5 knockout compared to 2D culture. (**A**) Venn diagrams generated on interactivenn.net represent significant upregulated transcriptome changes and (**B**) significant downregulated transcriptome changes in MDA-MB-231 parental vs. 2D^MDA-MB−231-ERK5-ko^, 3D spheroid^MDA-MB−231-ERK5-ko^, and BA-MaPS^MDA-MB−231-ERK5-ko^; (**C**) The number of significantly upregulated and downregulated genes was determined in 2D^MDA-MB−231-ERK5-ko^, 3D spheroid^MDA-MB−231-ERK5-ko^, and BA-MaPS^MDA-MB−231-ERK5-ko^ compared to the parental (p_adj_ ≤ 0.05). *n* = 3 biological replicates for 2D and 3D spheroid culture and *n* = 4 biological replicates for BA-MaPS.

To assess differences in ERK5-induced changes in gene expression across TNBC models, global transcriptomics analysis was performed on MDA-MB-231 parental and ERK5-ko cells cultured in 2D (2D^parental^/2D^MDA-MB−231-ERK5-ko/HS578T-ERK5-ko^), 3D spheroids (3D^parental^/3D^MDA-MB−231-ERK5-ko^), and BA-MaPS. Gene expression was normalized to parental lines for each model. There was limited overlap in the ERK5-ko transcriptome among the three culture models, where only 91 upregulated and 73 downregulated genes were commonly shared across the three systems (Figure 1A). We then evaluated pathways associated with genes that were enhanced by ERK5-ko across all three systems (2D culture, 3D spheroid, and BA-MaPS). Apical surface protein pathway was significantly upregulated (p_adj_ = 1.72 × 10^−2^), and estrogen response late (p_adj_ = 3.68 × 10^−2^) and k-RAS signaling late (p_adj_ = 3.68 × 10^−2^) pathways were significantly downregulated (Supplemental Table S2) in all three models. Transcripts in the estrogen response late pathway (BATF, OPN3, and AGR2) and k-RAS signaling (PCSK1N, ITGB2, and AGR2) were consistently reduced in all three models (Supplemental Figure S1). In both 2D^MDA-MB−231-ERK5-ko^ and BA-MaPS^MDA-MB−231-ERK5-ko^, Wnt-β Catenin (p_adj_ = 8.89 × 10^−3^) and Notch signaling pathways (cellular proliferation and migration) (p_adj_ = 2.40 × 10^−2^) were significantly downregulated (*p* < 0.05) (Supplemental Table S3) [6].

Compared to parental lines, there were more upregulated transcripts than downregulated in 2D^MDA-MB−231-ERK5-ko^ culture, while the number of downregulated genes was higher than upregulated genes in BA-MaPS^MDA-MB−231-ERK5-ko^ and 3D spheroid^MDA-MB−231-ERK5-ko^ conditions (Figure 1B). The number of differentially expressed genes was highest in 2D culture, with 2759 upregulated and 2391 downregulated genes. In comparison, 3D spheroids had a total of 768 upregulated and 698 downregulated genes, and BA-MaPS had a total of 326 upregulated and 698 downregulated genes (Figure 1C).

**Figure 2:**
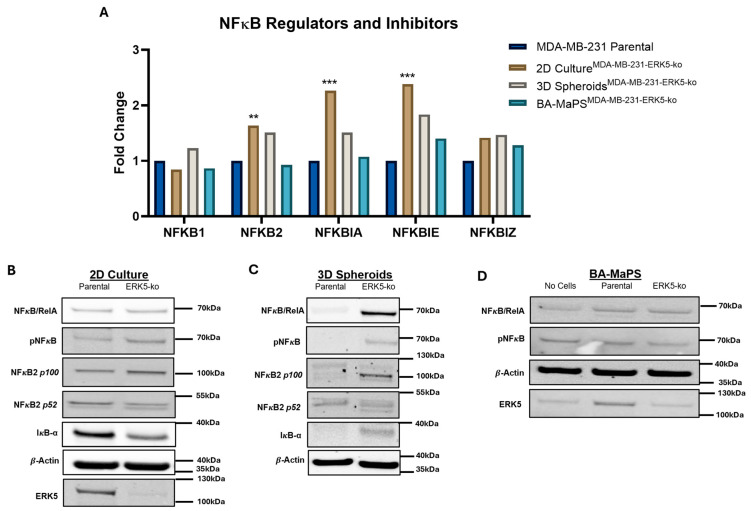
NFкB is upregulated in ERK5-ko cells when cultured in 2D. (**A**) RNA sequencing analysis of NFкB regulators and inhibitors (NFкB1, NFкB2, NFкBIA, NFкBIE, and NFкBIZ) in MDA-MB-231 parental and 2D^MDA-MB−231-ERK5-ko^ (*n* = 3), 3D spheroid^MDA-MB−231-ERK5-ko^ (*n* = 3), and BA-MaPS^MDA-MB−231-ERK5-ko^ (*n* = 4). Data represent mean ± SEM of triplicate (2D culture and 3D culture) and quadruplicate (BA-MaPS) experiments. ** *p* ≤ 0.01 and *** *p* ≤ 0.001. (**B**) Western blot analysis of MDA-MB-231 parental and ERK5-ko cell lysates cultured in 2D, where *n* = 3 biological replicates. (**C**) MDA-MB-231 parental and ERK5-ko 3D spheroids were formed in an Aggrewell 24-well plate, and Western blot was performed on cell lysates, where *n* = 3 biological replicates. (**D**) MDA-MB-231 parental and ERK5-ko cells were cultured in BA-MaPS for 5 days. Western blot was performed on cell and tissue lysates, where *n* = 3 biological replicates. The uncropped blots are shown in Figure S8.

We further analyzed differentially expressed genes in ERK5-ko systems and identified the top five significantly altered KEGG pathways (*p* < 0.005) for each culture model (Table 1) through Enrichr gene set analysis database. In 2D^MDA-MB−231-ERK5-ko^ culture, significantly upregulated genes were associated with NFκB and inflammation (TNFα signaling via NFκB, hypoxia, apoptosis, glycolysis, and IL2/STAT5 signaling). ERK5 depletion decreased the expression of cell proliferation-related genes (MYC targets V1 and MYC targets V2). In 3D spheroid^MDA-MB−231-ERK5-ko^, genes linked to EMT, E2F targets, and G2-M checkpoint were upregulated, while genes involved in glycolysis and P53 signaling were downregulated. In BA-MaPS^MDA-MB−231-ERK5-ko^, expression of apical surface protein markers were elevated, and transcripts related to proliferation pathways, k-RAS signaling, and NFkB signaling were reduced. As genes associated with TNFα-NFκB signaling were disproportionally regulated between the culture models, we evaluated specific transcripts in these pathways. Expression of NFκB-related genes JUNB and BHLHE40 was increased in 2D culture but decreased in BA-MaPS (Supplemental Figure S2). Additionally, expression of NFκB2, NFкBIA/IκBα, and NFκBIE/IκBε increased significantly (p_adj_ < 0.05) in 2D^MDA-MB−231-ERK5-ko^ cells, but this was not observed in 3D spheroid^MDA-MB−231-ERK5-ko^ nor BA-MaPS^MDA-MB−231-ERK5-ko^ (Figure 2A).

### 3.2. NFκB Is Selectively Upregulated in ERK5-ko Cells When Cultured in 2D and 3D Spheroids, Compared to BA-MaPS

Based on differences in NFκB-associated genes across the models in ERK5-ko cells, we next assessed activation of NFκB components (Supplemental Table S4) in 2D (Figure 2B, Figures S4C and S8A,B), 3D spheroid (Figure 2C, Figures S4D and S8C,D), and BA-MaPS (Figure 2D, Figures S4E and S8E). In 2D culture, levels of phosphorylated NFκB p65/RelA and total NFκB2 p100 were significantly upregulated (*p* < 0.001) with loss of ERK5 in MDA-MB-231 cells but not in Hs578T cells (Supplemental Figures S4A,C and S8B), suggesting cell line-specific effects. Consistent with results in 2D, NFκB2 p100 protein levels were significantly upregulated (*p* < 0.05) in 3D Spheroid^MDA-MB−231-ERK5-ko^ compared to parental spheroids (Supplemental Figure S4D). Expression of the p52 active form of NFκB2 was also elevated in this model (*p* < 0.05). Interestingly, NFκB total and phosphorylated protein levels were comparable in BA-MaPS^MDA-MB−231^ and BA-MaPS^MDA-MB−231-ERK5-ko^ (Figure 2D, Figures S4E and S8E).

### 3.3. ERK5 Modulates TNBC Motility in 2D, 3D Spheroid, and BA-MaPS Cultures

**Figure 3:**
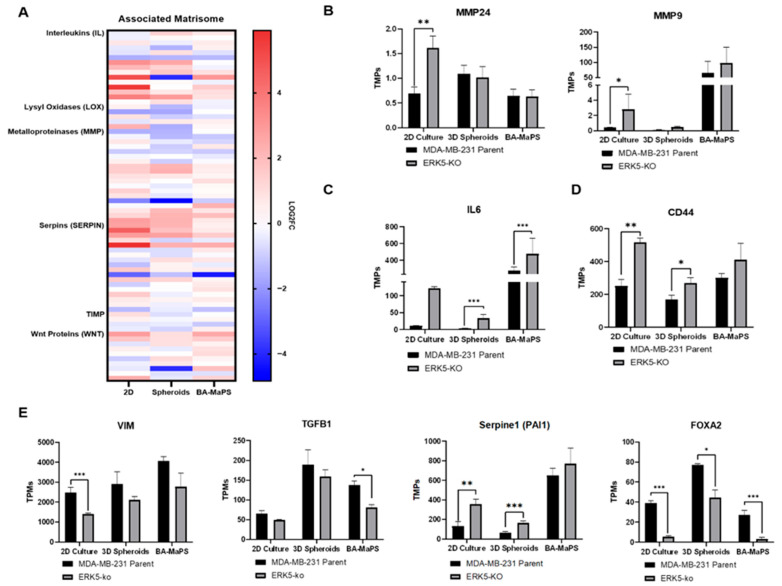
Culture models impact ERK5 regulation of EMT and motility-related genes. (**A**) Heat map of associated matrisome genes, defined by the MIT Matrisome Project, upregulated and downregulated in 2D^MDA-MB−231-ERK5-ko^, 3D spheroid^MDA-MB−231-ERK5-ko^, and BA-MaPS^MDA-MB−231-ERK5-ko^. Gene changes are represented by log 2-fold change in significant genes normalized to corresponding parental control. (**B**) Transcripts per million (TPMs) of matrix metalloproteinase genes MMP24 and MMP9, (**C**) inflammation marker IL-6, (**D**) migration/cell adhesion marker CD44, and (**E**) EMT-related genes VIM, TGFB1, SERPINE 1 (PAI1), and FOXA2. Data represent mean ± SEM of at least 3 independent experiments. * *p* ≤ 0.05, ** *p* ≤ 0.01, and *** *p* ≤ 0.001. *n* = 3 biological replicates in 2D and 3D spheroid culture and *n* = 4 in BA-MaPS.

Previous work by our group and others has demonstrated ERK5 regulation of the TME. Due to the discrepancies in intracellular signaling pathways, we next explored the impact of ERK5 on genes associated with TME interactions. Across the three culture systems, genes related to inflammation (interleukins), migration (lysyl oxidases), and EMT (metalloproteinases, serpines, tissue inhibitor of metalloproteinases, and Wnt proteins) were differentially expressed in MDA-MB-231-ERK5-ko cells compared to parental cells (Figure 3A). Notably, these genes all correlate with remodeling of the extracellular space and cell motility. Matrix metalloproteinase genes MMP24 and MMP9 were significantly upregulated (p_adj_ < 0.002; p_adj_ < 0.05) in 2D^MDA-MB−231-ERK5-ko^ culture compared to the parental. These MMPs were not altered in the 3D spheroid^MDA-MB−231-ERK5-ko^ or BA-MaPS^MDA-MB−231-ERK5-ko^ models (Figure 3B). Expression of inflammation marker IL6 was elevated in 2D^MDA-MB−231-ERK5-ko^ (p_adj_ = 2.14 × 10^−75^) and 3D spheroid^MDA-MB−231-ERK5-ko^ compared to parental cell lines, but not in BA-MaPS^MDA-MB−231-ERK5-ko^ (Figure 3C). While cancer stem cell marker CD44 and EMT-related genes linked to metastasis (VIM, TGFB1, and PAI1/SERPINE1) were variable across the three systems (Figure 3D,E), transcript levels of forkhead box (FOX) family member FOXA2 were lower in ERK5-ko conditions compared to parental control in all three culture systems, suggesting that FOXA2 is a direct target of ERK5 (p_adj_ = 9.789 × 10^−32^ for 2D, p_adj_ < 0.002 for 3D spheroid, and p_adj_ < 0.0002 for BA-MaPS).

**Figure 4:**
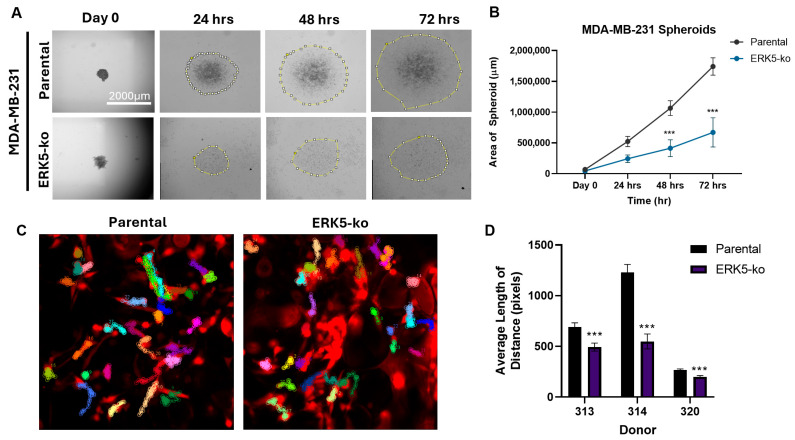
ERK5 increases motility in TNBC cells when cultured in 3D spheroids and BA-MaPS. (**A**) Representative images of MDA-MB-231 parental and ERK5-ko spheroid pseudo-migration assay after 72 h. (**B**) Spheroid area was quantified after 24 h, 48 h, and 72 h in Fiji/ImageJ and normalized to Day 0. Experiments were completed in biological triplicate ± SEM and analyzed using two-way ANOVA and Sidak’s multiple comparison test. (**C**) Cell movement and activity of BA-MaPS^MDA-MB−231-ERK5-ko^ and BA-MaPS^MDA-MB−231-parental^ were recorded by time-lapse imaging, with representative images shown. Colored tracks represent 30 random individual cellular pathways traveled per frame. (**D**) Cell tracking analysis was completed on ImageJ add-on MTrackJ. Data represent average length of 12–31 random MDA-MB-231 parental and ERK5-ko cells in *n* = 3 different breast tissue donors (313, 314, and 320) ± SEM, paired *t*-test. *** *p* ≤ 0.001.

To assess ERK5 regulation of motility, we performed model-specific migration assays for 2D culture, 3D spheroid, and the BA-MaPS using ERK5-ko cells and compared the response to parental cell lines. We have previously shown and re-validated that ERK5 depletion inhibits migration in the TNBC cell lines MDA-MB-231 and Hs578T in 2D culture (Supplemental Figure S3) [6]. Using a pseudo-migration assay to analyze 3D spheroids, we observed significantly decreased migration of MDA-MB-231-ERK5-ko cells (Figure 4A,B) and Hs578T-ERK5-ko cells (Supplemental Figure S5) (*p* < 0.001) compared to parental cells after 48 h and 72 h in culture. Cancer cell motility in BA-MaPS was determined through fluorescent cancer cell tracking by time-lapse imaging. BA-MPS models were derived from three individual donor breast tissues (Supplemental Table S1), and in all three tissues, MDA-MB-231-ERK5-ko cells migrated less than parental cells (492.8 vs. 691.2 px. in donor 313; 548.1 vs. 1230 px in donor 314.; 199.2 vs. 265.9 px. in donor 320) (*p* < 0.05) (Figure 4C,D and Figure S6). We used a different confocal microscope for time-lapse analysis of Hs578T parental and ERK5-ko cells cultured in BA-MaPS, and through the SlideBook software, particle tracking analysis was used to quantitatively determine average speed and displacement, rather than the length of distance. Consistent with results in 2D and 3D, ERK5 ablation decreased the motility of Hs578T cells in BA-MaPS, with an average speed of ~0.002 μm/s and average total displacement of 10.7 μm for ERK5-ko compared to parental cells (0.0037 μm/s and 16.77 μm, respectively) (*p* < 0.001) (Supplemental Figure S7).

### 3.4. ERK5 Modulates Core Matrisome Profiles Differently in 2D, Spheroid, and BA-MaPS Cultures

**Figure 5:**
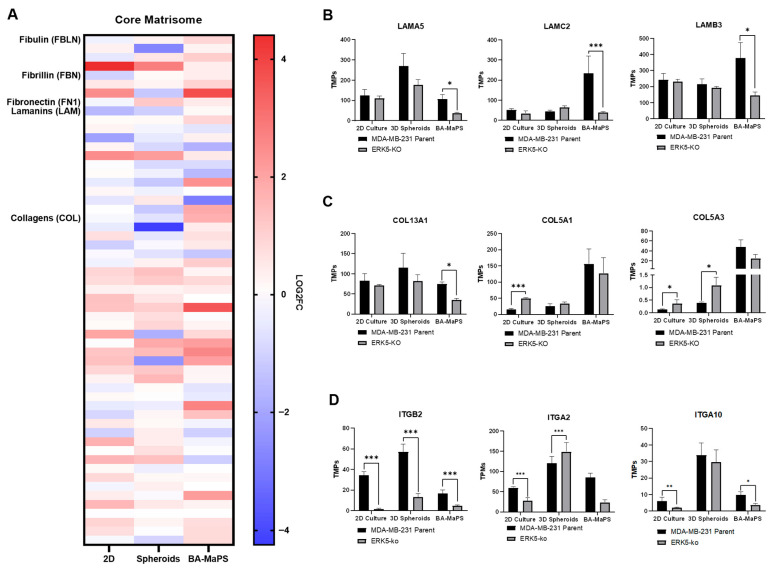
ERK5 modulates extracellular matrix genes differently in 2D culture, 3D spheroids, and BA-MaPS. (**A**) Heat map of core matrisome genes, defined by MIT Matrisome Project, upregulated and downregulated in 2D^MDA-MB−231-ERK5-ko^, 3D spheroid^MDA-MB−231-ERK5-ko^, and BA-MaPS^MDA-MB−231-ERK5-ko^. Gene changes are represented by log 2-fold change in significant genes (p_adj_ ≤ 0.05) normalized to 2D^MDA-MB−231-parental^, 3D spheroid^MDA-MB−231-parental^, and BA-MaPS^MDA-MB−231-parental^. (**B**) Transcripts per million (TPMs) of laminins, (**C**) collagens, and (**D**) integrins. Data represent mean ± SEM of *n* = 3 biological replicates in 2D culture, *n* = 3 in 3D spheroid culture, and *n* = 4 in BA-MaPS experiments. * *p* ≤ 0.05, ** *p* ≤ 0.01, and *** *p* ≤ 0.001.

Based on motility differences and changes in the associated matrisome, we then determined alterations in core ECM. Using RNA sequencing data, we compared core matrisome gene signature (fibulins, fibrillins, fibronectin, laminins, and collagens) in MDA-MB-231 parental and ERK5-ko cells cultured in 2D, 3D spheroids, and BA-MaPS (Figure 5A). Compared to parental cells, laminin components LAMA5, LAMC2, and LAMB3 decreased in ERK5-ko cells only in BA-MaPS (p_adj_ < 0.05) (Figure 5B). Expression of collagen genes varied among the systems in ERK5-null cells compared to parental MDA-MB-231 cells. Specifically, collagen type V genes were upregulated with loss of ERK5 (COL5A1 and COL5A3 in 2D, p_adj_ < 0.05, and COL5A3 in 3D spheroids, p_adj_ < 0.01) (Figure 5D). In contrast, collagen transcript levels in ERK5-ko cells were comparable to those of parental control in BA-MaPS, with the only significant downregulation in COL13A1 expression (p_adj_ = 0.0118) (Figure 5C). Consistent with changes in ECMs, ECM receptors were also altered across the three models. Gene expression of integrins (ITGB2, ITGA2, and ITGA10) was reduced in 2D and BA-MaPS ERK5-ko models (p_adj_ < 0.05). Only ITGB2 was downregulated in ERK5-ko 3D spheroids (p_adj_ < 0.001) (Figure 5D).

### 3.5. ERK5 Alters Breast Tissue ECM Composition

**Figure 6:**
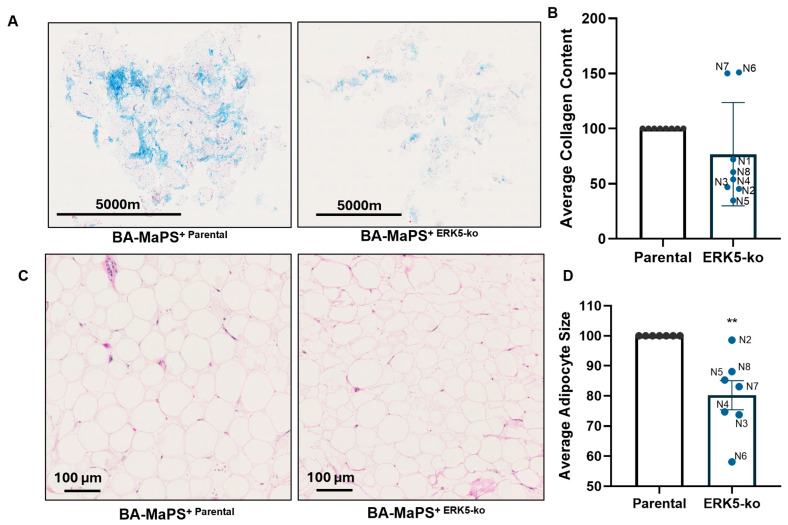
ERK5 affects breast tissue architecture in BA-MaPS. (**A**) Representative images of Masson’s trichrome stains of BA-MaPS^MDA-MB−231-ERK5-ko^ and BA-MaPS^MDA-MB−231-Parental^ lines after 5 days of culture (objective imaged at 20X). (**B**) BA-MaPS^MDA-MB−231-ERK5-ko^ and BA-MAPS^MDA-MB−231-Parental^ lines were analyzed on Inform Tissue Analysis Software by Akoya Biosciences for average collagen content. (**C**) Representative H&E staining of BA-MaPS^MDA-MB−231-ERK5-ko^ and parental lines. (**D**) Adipocyte size in px was analyzed in BA-MaPS^MDA-MB−231-ERK5-ko^ and parental lines. Data represent mean ± SEM of 8 different donors. ** *p* ≤ 0.01.

Changes in ECM expression and secreted cytokines (IL-6 and TGFB) suggest remodeling of the TME. While this cannot be investigated in 2D or 3D models, our BA-MaPS model provides a platform to define ERK5 contribution to TME modification, specifically effects on collagen content and adipocyte size. Collagen expression was evaluated in BA-MaPS from eight different patient donor tissues, experiments N1-N8 (Supplemental Table S1), by Masson’s trichrome stain (Figure 6A). Collagen content in BA-MaPS cultured with MDA-MB-231-parental cells was not significantly different from that of ERK5-ko cells (Figure 6B).

To further delineate ERK5-mediated changes in the TME, we assessed adipocyte size through H&E. ERK5-ko significantly reduced average adipocyte size in BA-MaPS compared to parental control (*p* < 0.01) (Figure 6C,D), suggesting that ERK5 plays a role in adipocyte hypertrophy and increasing adipocyte dysfunction [23].

## 4. Discussion

Prior studies, including our previous work, have delineated ERK5 regulation of EMT in vitro as well as tumor growth, architecture, and ECM composition in vivo [6,24,25,26]. Here, we expanded upon our research in 2D and examined the impacts of culture models on ERK5-mediated effects. Although pathways varied in each system, ERK5 depletion decreased expression of genes involved in cell proliferation, particularly k-RAS signaling in all three models (Supplemental Table S2). Downregulated proliferation-associated pathways in the culture conditions include MYC targets in V1 and V2 in 2D culture, p53 signaling in 3D spheroids, and k-RAS in BA-MaPS. These transcript and pathway changes are aligned with prior works showing ERK5-mediated breast cancer cell proliferation [27,28,29].

The NFκB pathway, a known downstream effector of ERK5, regulates inflammation, proliferation, differentiation, and apoptosis, and is found to be abnormally expressed and activated in breast cancers [30,31,32]. In addition, cancer chemotherapy and radiation can activate NFκB signaling, promoting treatment resistance [33]. The NFκB family of transcription factors includes five members associated with transcriptional activation of NFκB: RelA (p65), C-Rel, RelB, NFκB1 p50, and NFκB2 p52. These five subunits are responsible for the transcriptional activation of NFκB, while the p50 and p52 precursors, p105 and p100, along with IκBα, IκBβ, and IκBε proteins, are involved in the inhibition of NFκB [33,34]. NF-κB mediates JUNB transcription, a key regulator of VEGF, and consequently, tumor angiogenesis in vivo, based on analysis of vascularization in JunB-deficient teratocarcinomas compared to wild-type tumors [35].

While NFκB components were not upregulated in BA-MaPS^MDA-MB−231-ERK5-ko^, JUNB levels were diminished in this system (Supplemental Figure S2), suggesting mechanistic links between JUNB and ERK5 in the regulation of angiogenesis [6,36]. In contrast with BA-MaPS data, JUNB expression is elevated in 2D-cultured ERK5-ko cells compared to the MDA-MB-231 parental line, in concordance with immunoblot analysis showing enhanced phosphorylation of NFκB/RelA (p65) and decreased expression of NFκB inhibitor IκBα in ERK5-depleted cells. Levels of phosphorylated NFκB were not elevated in Hs578T-ERK5-ko cells compared to parental control in 2D culture, denoting cell line-specific effects on ERK5-induced NFκB activation. While both cell lines are classified as Basal B, there is evidence that Hs578T is a metaplastic breast cancer cell line, a subset that is highly refractory to treatment [19,37,38]. Further studies are needed to evaluate the role of ERK5 signaling in this therapy-resistant breast cancer subtype. Upregulation of NFκB in ERK5-null MDA-MB-231 cells suggests compensatory activation of NFκB through alternative signaling. In BA-MaPS, cell-tissue interactions may maintain NFκB signaling in the absence of ERK5, accounting for comparable levels of NFκB phosphorylation in parental and ERK5-null cells. Additionally, broader proteomic analysis of these models may provide insight on activation of NFκB components that were not evaluated in our panel. In a study using lung fibroblasts cultured in 2D and 3D scaffolds, NFκB activation was higher in 2D, but the activity threshold is higher in 3D [39], supporting further analysis of culture-dependent effects. Elucidating the dynamics of NFκB signaling in our systems would improve understanding of this pathway for therapeutic targeting.

While there was model-based variability in ERK5-mediated effects, loss of ERK5 impaired migration significantly across all three systems. Our transcriptomic analysis shows suppression of EMT-related genes in ERK5-ko cell lines, further establishing ERK5 regulation of EMT [40]. Expression of EMT genes TGFβ1, VIM, and PAI1 varied among culture systems, but FOXA2 was significantly downregulated in all models (Figure 3), implicating its role in the ERK5-EMT axis. To our knowledge, links between ERK5 and FOXA2 have not been investigated, but there are known overlaps in their interactors and impact on cellular processes, such as proliferation and differentiation, contributing to the cancer stem cell phenotype [24,41,42,43,44,45]. The role of FOXA2 in TNBC biology is not clearly defined. Overexpression of FOXA2 suppressed MDA-MB-231 proliferation and tumor growth [46], but there is also evidence linking FOXA2 mRNA expression with significantly higher recurrence rates in TNBC patients [42]. Consistent with the latter, siRNA-mediated knockdown of FOXA2 reduced TNBC mammosphere formation and cancer stem cell proliferation in vitro [42]. Our results support these findings, as loss of ERK5 and subsequent downregulation of FOXA2 reversed the EMT phenotype in TNBC models. Due to the paradoxical effects of FOXA2, further research is needed to clarify its function in tumor formation and progression.

In addition to identifying ERK5 regulation of cell motility across a series of complex in vitro models, we also demonstrate ERK5 as a mediator of cell extrinsic signaling and the TME. The extracellular matrix can promote TNBC growth, migration, and invasion through altered stiffness and signaling components such as integrins, laminins, and collagens [47]. Previously, we showed that ERK5 regulates many ECM components [6]. ERK5-ko did not change major collagen fibers (COL I/III), consistent with Masson’s trichrome stain of collagen fibers. These findings are consistent with prior reports by our group and others, highlighting that major fibril collagens are not altered in TNBC compared to other breast cancer subtypes [48,49]. Loss of ERK5 in 2D and 3D increased the expression of minor fibril collagen and collagen type V gene expression, while COL13A1 was repressed in BA-MaPS-ERK5-ko models (Figure 5C). COL13A1 is elevated in TNBC and positively correlates with tumor progression and metastasis [50,51]. TNBC modeling in 3D using ECM scaffolds demonstrates laminin-induced vascularization. While not specifically evaluated here, a potential mechanism by which ERK5-ko inhibits the aggressive phenotype of TNBC is through regulation of ECM remodeling, specifically repressing ECMs required for stromal induction of vascularization. Further, laminins that are significantly downregulated in BA-MaPS^MDA-MB−231-ERK5-ko^ LAMC2 and LAMA5 are involved in EMT and cancer cell proliferation, respectively [52,53]. ERK5 ablation in TNBC cells significantly decreased expression of integrins across all models, although transcript levels of specific integrins varied in each system [54]. Additional TME remodeling was evident by decreased adipocyte size with loss of ERK5 expression. Prior studies comparing 2D models demonstrate the loss of ECMs and TME factors in conventional cancer cell culture models. As our model system advances from 2D, 3D, and BA-MaPS, we observed alterations to the regulation of ECM factors with ERK5-ko. As ECM modifies cancer cell function, such as response to therapy, proliferation, and EMT [55,56,57,58], culture models that fully integrate these factors are critical. Further, evaluating the interplay between ERK5 and ECM may provide novel therapeutic strategies.

Our data highlight the intricacies of signaling networks that vary among cancer model systems. Notably, expression of IL6 was upregulated in ERK5-ko cells in 2D and 3D compared to parental cell lines, but IL6 levels were comparable in the BA-MaPS between parental and ERK5-ko conditions (Figure 3C), potentially due to higher baseline expression of this adipokine in the BA-MaPS compared to 2D and 3D models. The use of obese breast tissue in our macrophysiological system [59,60] may also contribute to elevated IL6 levels. Thus, the ERK5-IL6 axis is culture-dependent, denoting a need to define the impact and origin of protumorigenic secreted factors from both cancer cells and stromal cells.

## 5. Conclusions

The development and use of 3D macrophysiological models will increase accuracy in breast cancer research, accelerating validation of novel therapeutics. Through the BA-MaPS, we further defined the role of ERK5 and ECM, EMT, and migration and characterized cell–cell and cell-tissue interactions in TNBC. The variable results among 2D culture, 3D spheroid, and BA-MaPS stress the importance of using physiologically relevant models in breast cancer research. Our findings provide a basis for future mechanistic studies on the ERK5-FOXA2 axis and ERK5 interplay with NFκB signaling and in targeting TNBC.

## Figures and Tables

**Table 1:** Pathways associated with the ERK5-ko transcriptomic signature compared to parental control in MDA-MB-231 cells cultured in 2D, 3D spheroids, and BA-MaPS (Hallmarks Database).

Model	Upregulated Genes		Downregulated Genes	
	Pathway	*p*-value	Pathway	*p*-value
Two-dimensional culture	TNFα Signaling via NFκB	3.33 × 10^−20^	G2-M Checkpoint	5.68 × 10^−10^
	Hypoxia	3.33 × 10^−20^	E2F Targets	3.57 × 10^−8^
	Apoptosis	1.25 × 10^−11^	Myc Targets V2	1.58 × 10^−6^
	Glycolysis	8.60 × 10^−11^	Myc Targets V1	8.25 × 10^−6^
	IL-2/STAT5 Signaling	5.78 × 10^−10^	Estrogen Response Late	0.001357
Three-dimensional spheroid	E2F Targets	4.6 × 10^−14^	Estrogen Response Early	3.9 × 10^−10^
	Interferon Gamma Response	3.43 × 10^−11^	Estrogen Response Late	8.16 × 10^−9^
	Epithelial–Mesenchymal Transition	7.47 × 10^−10^	Hypoxia	1.43 × 10^−7^
	k-RAS Signaling Up	7.99 × 10^−7^	Glycolysis	4.54 × 10^−3^
	G2-M Checkpoint	2.82 × 10^−6^	P53 Pathway	1.07 × 10^−2^
BA-MaPS	Apical Surface	5.63 × 10^−3^	k-RAS Signaling Up	5.29 × 10^−5^
	Estrogen Response Early	1.71 × 10^−2^	Estrogen Response Late	5.53 × 10^−4^
	Myogenesis	1.71 × 10^−2^	TNFα Signaling via NFкB	4.42 × 10^−3^
	Complement	1.71 × 10^−2^	Glycolysis	4.42 × 10^−3^
	Coagulation	2.57 × 10^−2^	Estrogen Response Early	2.63 × 10^−2^

## Data Availability

All data are contained within this manuscript and the Supplemental Documents. The RNA sequencing data discussed in this publication have been deposited in NCBI’s Gene Expression Omnibus, accessible through the GEO Series accession number GSE316523.

## Supplementary Materials

The following supporting information can be downloaded at https://www.mdpi.com/article/10.3390/cancers18030376/s1. Supplemental Figure S1: Overlapping upregulated and downregulated transcript in 2D culture, 3D spheroid, and BA-MaPS; Supplemental Figure S2: Overlapping modified genes in the NFκB pathway in 2D and BA-MaPS; Supplemental Figure S3: ERK5 migration analysis confirmation in 2D culture; Supplemental Figure S4: Western blot analysis of NFκB pathway members in MDA-MB-231 and Hs578T; Supplemental Figure S5: Hs578T 3D spheroid pseudo-migration assay; Supplemental Figure S6: Time-lapse migration assay in MDA-MB-231 parental and ERK5-ko cells seeded in BA-MaPS with additional donors outlined in Figure 5; Supplemental Figure S7: Time-lapse migration assay in Hs578T parental and ERK5-ko cells seeded in BA-MaPS; Supplemental Figure S8: Origional western blot images; Supplemental Table S1: Human breast tissue donor information; Supplemental Table S2: Transcriptomic significant pathways in MDA-MB-231-ERK5-ko cells cultured in all models; Supplemental Table S3: Transcriptomic significant pathways in MDA-Mb-231-ERK5-ko cells culture in 2D and BA-MaPS; Supplemental Table S4: NFκB transcript and protein names and function.

## Abbreviations

The following abbreviations are used in this manuscript:

ANOVA	One-Way Analysis Of Variance
ATCC	American Type Culture Collection
BA-MaPS	Breast Adipose-Macrophysiological System
Bp	Base Pair
brASC	Breast Adipose Stromal Cell
ECM	Extracellular Matrix
EMT	Epithelial-to-Mesenchymal Transition
ERK5	Extracellular Signal-Regulated Kinase 5
FACS	Fluorescent-Activated Cell Sorting
HER2	Human Epidermal Growth Factor Receptor 2
MAPK7	Mitogen-Activated Protein Kinase 7
MPER	Mammalian Protein Extraction Reagent
NACT	Neoadjuvant Chemotherapy
PCR	Polymerase Chain Reaction
TBS-T	Tris-Buffered Saline
TNBC	Triple-Negative Breast Cancer
