# Supplementary material for: Dissecting Context-Specific Effects of ERK5 Signaling in Triple-Negative Breast Cancer
Source: Cancers (Basel). 2026 Jan 26;18(3):376. doi: 10.3390/cancers18030376 (PMC12896816; doi:10.3390/cancers18030376)

Supplemental Figure S8A1-6: Original western blots and ImageJ analysis of MDA-MB-231 parental and ERK5-ko cells cultured in 2D. Cropped western blots from 1-6 are shown in Figure 2B.

1. <u>IκBα – IRDye 800CW</u> :

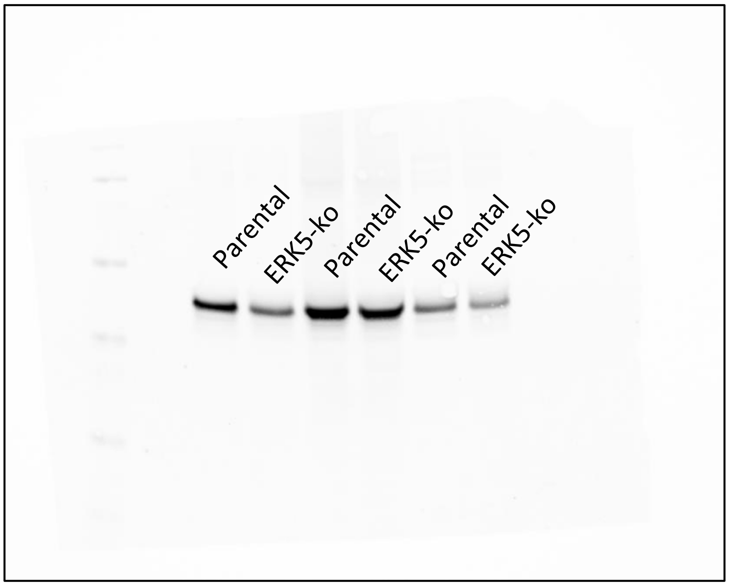

ImageJ analysis

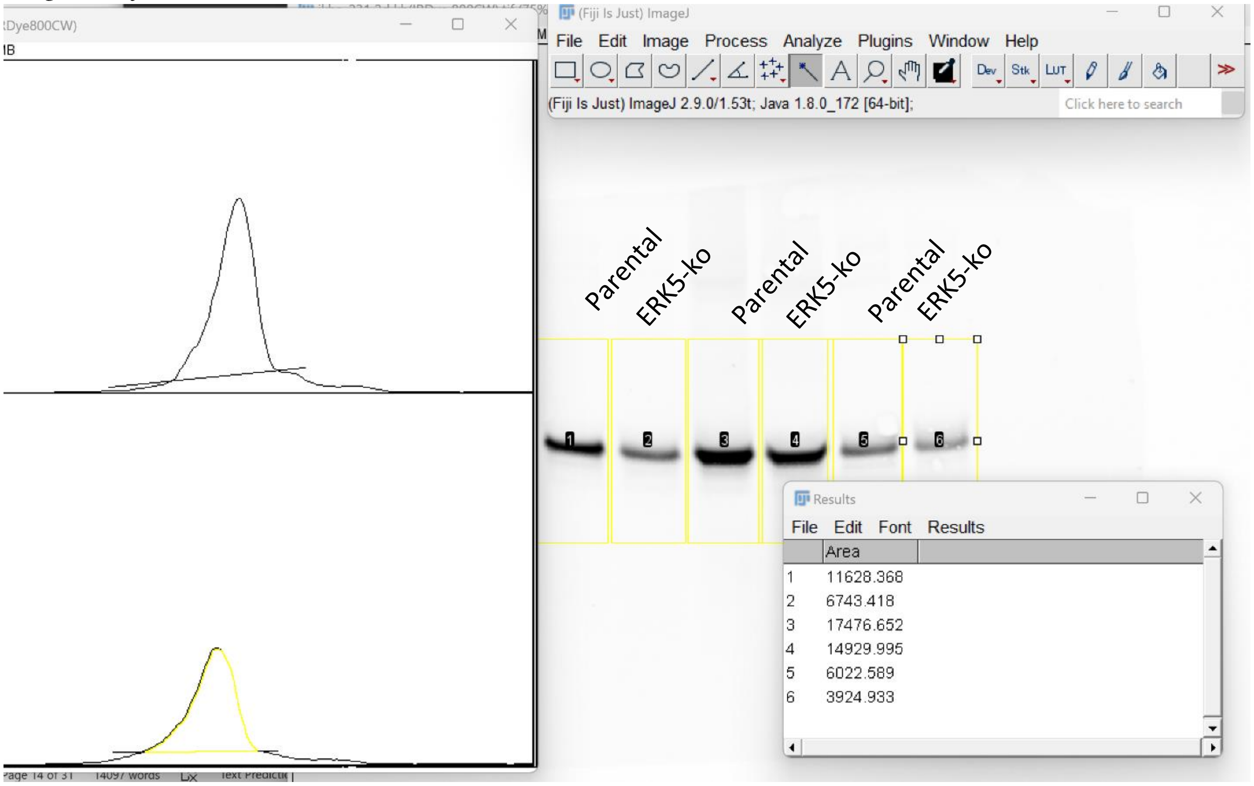

2. <u>ERK5 – IRDye 680RD</u>

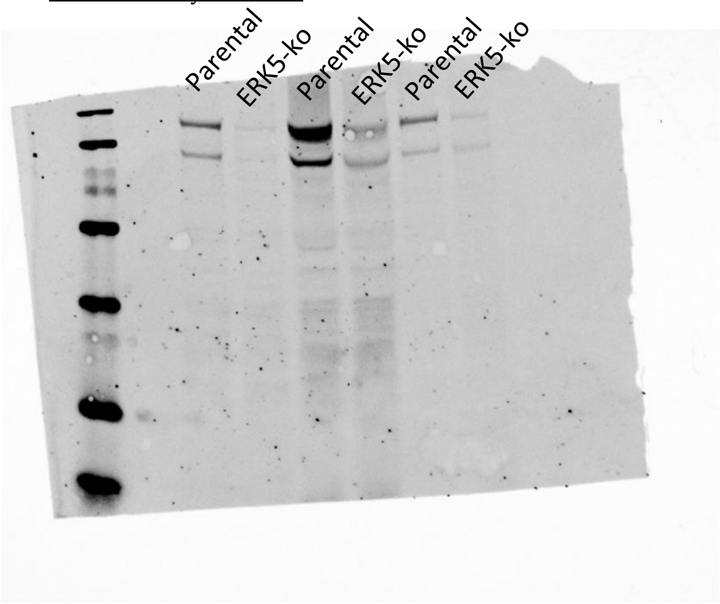

ImageJ Analysis

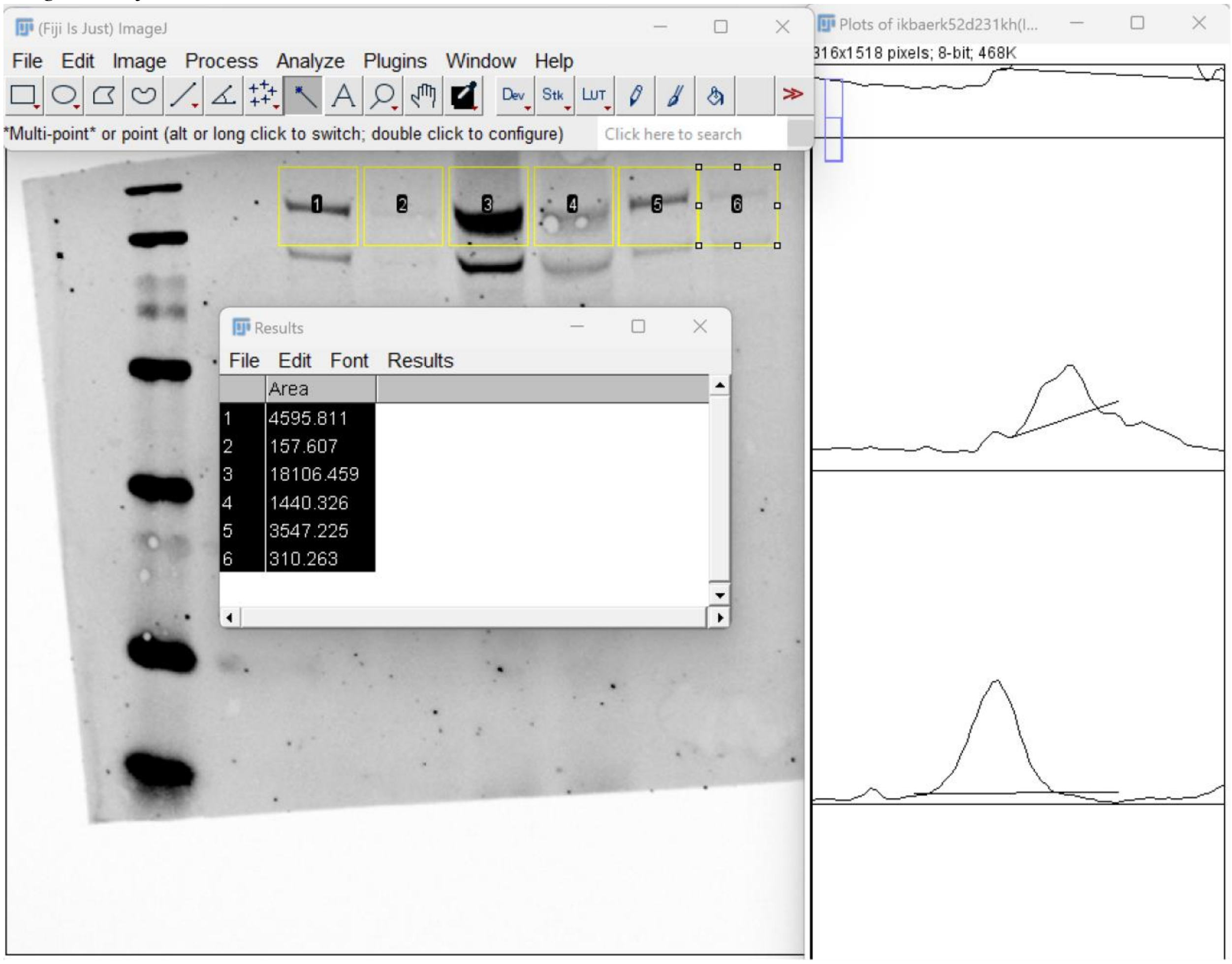

3. <u>NFκB/RelA – IRDye 800CW</u>

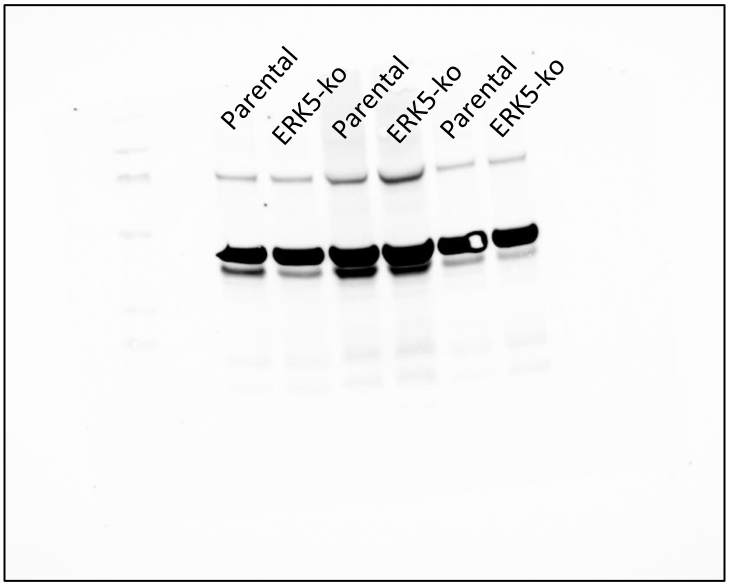

ImageJ Analysis

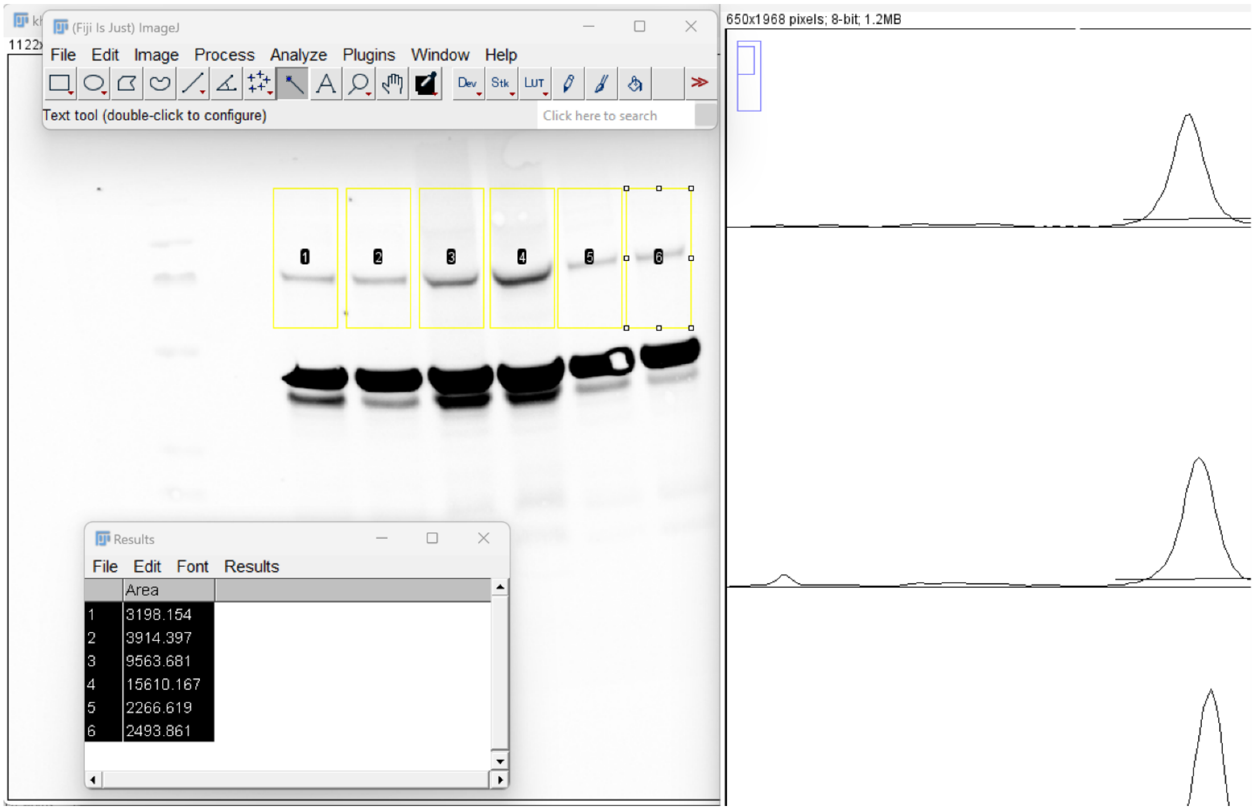

4. pNFκB – IRDye 680RD

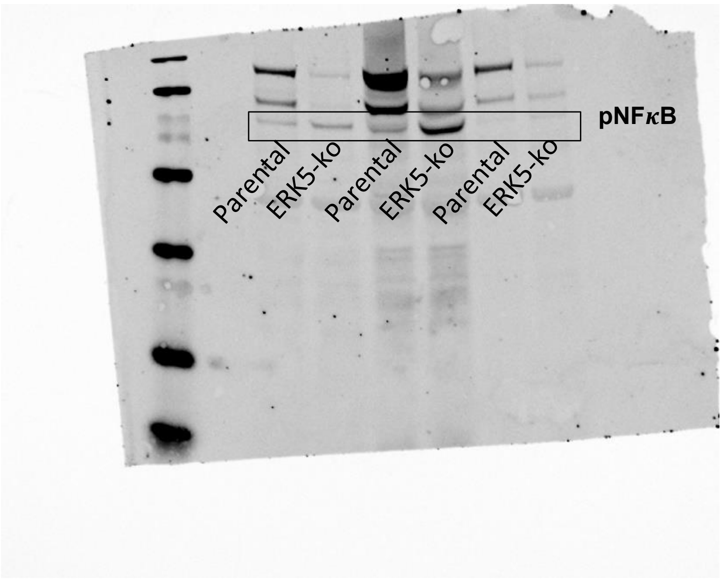

ImageJ Analysis

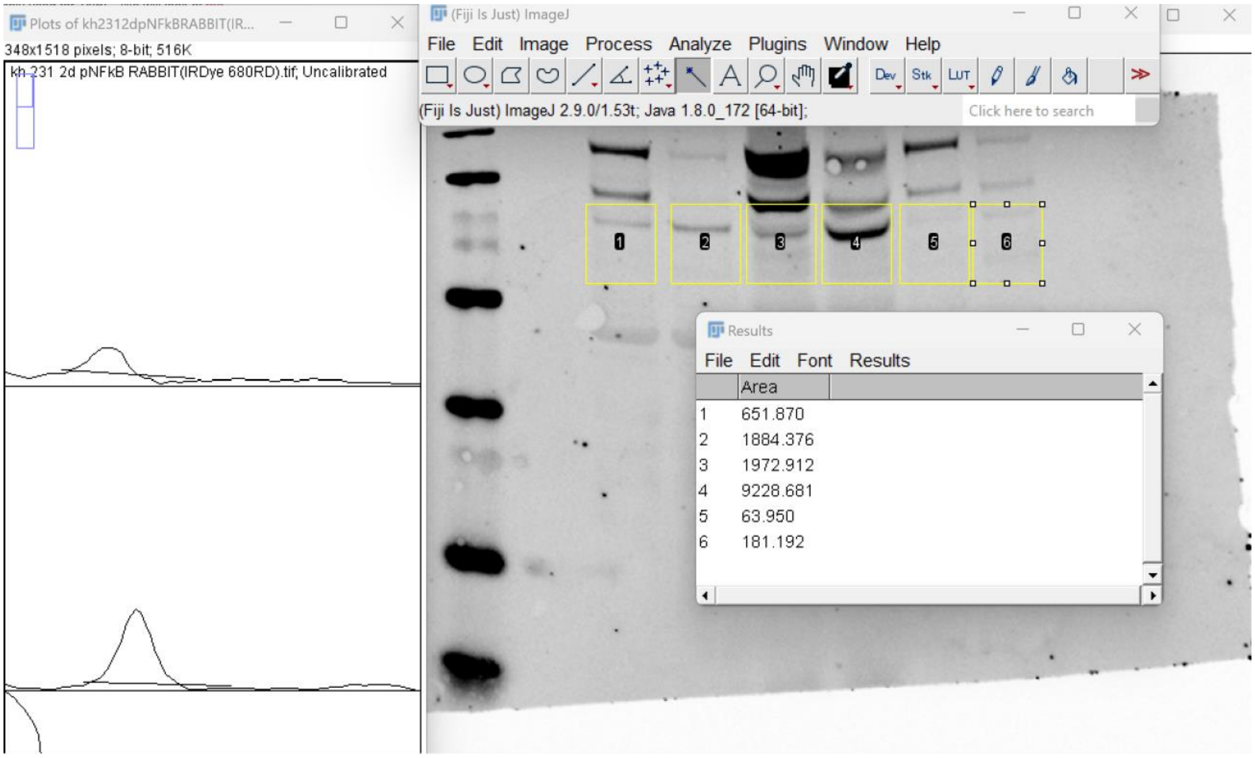

5. <u>NFκB2 (p100 and p52) – IRDye 680RD</u>

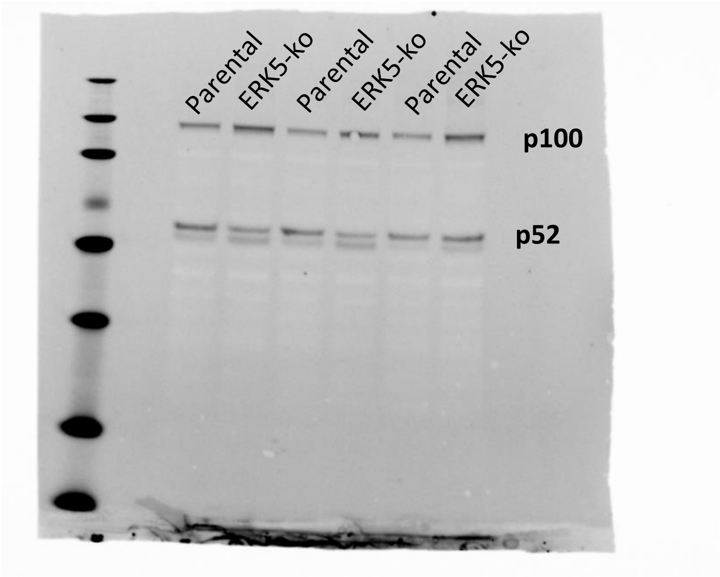

ImageJ Analysis

p100

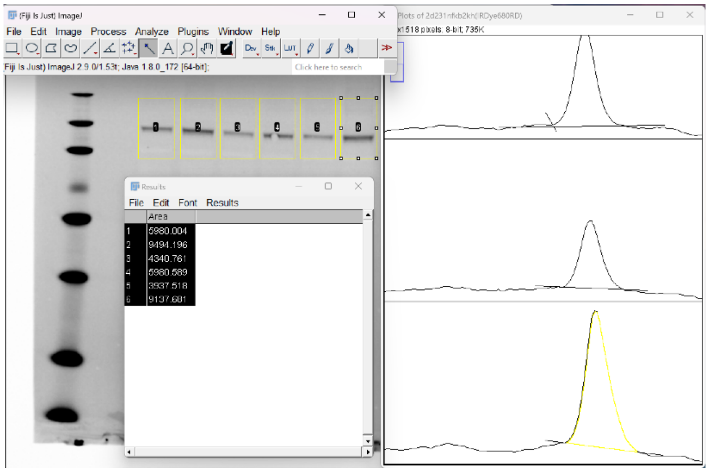

p52

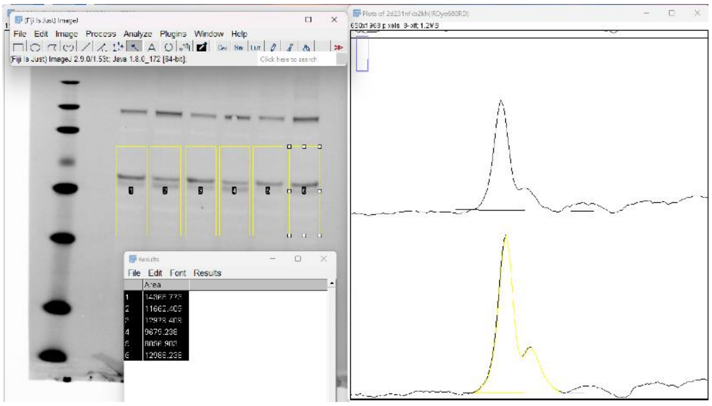

6. <u>β-Actin – IRDye 800CW</u>

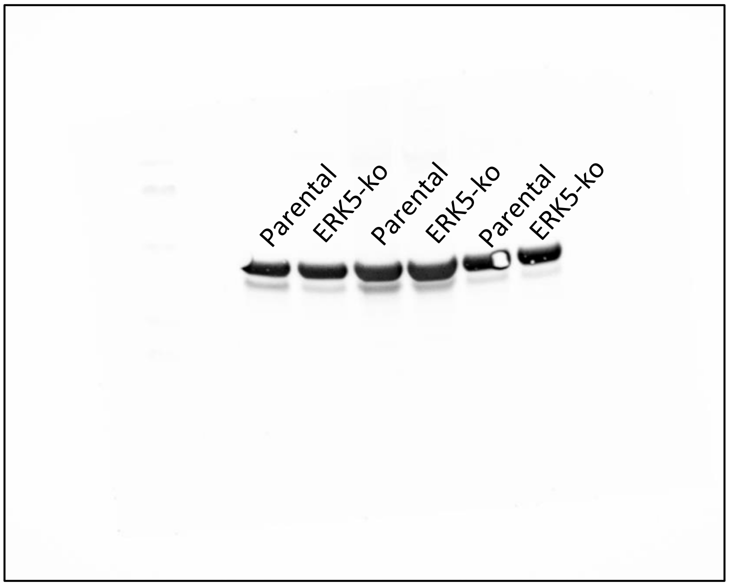

ImageJ Analysis

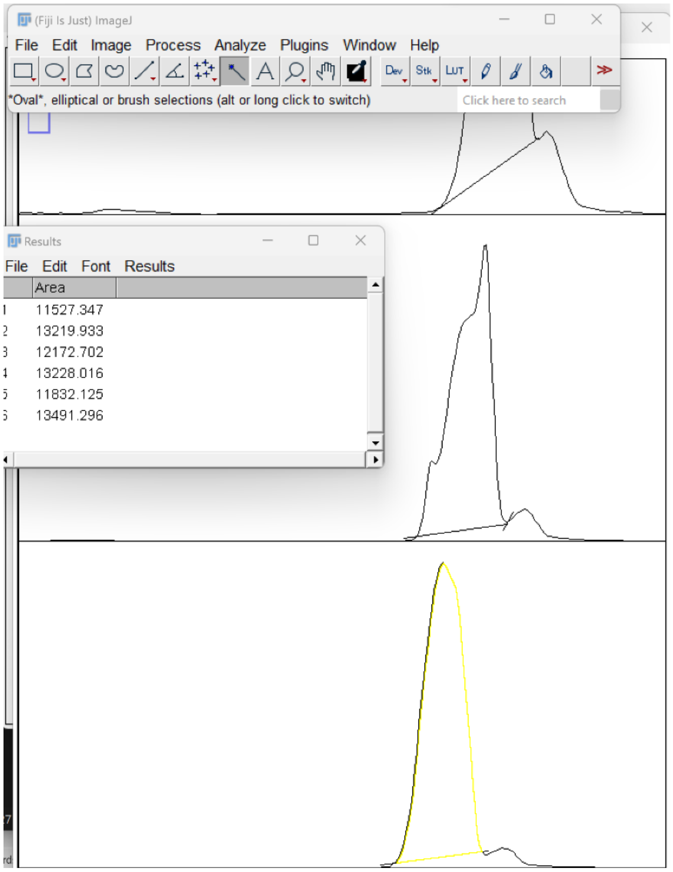

Supplemental Figure S8B1-5: Original western blots and ImageJ analysis of Hs578T parental and ERK5-ko cells cultured in 2D. Cropped western blots from 1-5 are shown in Supplemental Figure S4A.

1. <u>ERK5 – IRDye 680RD</u>

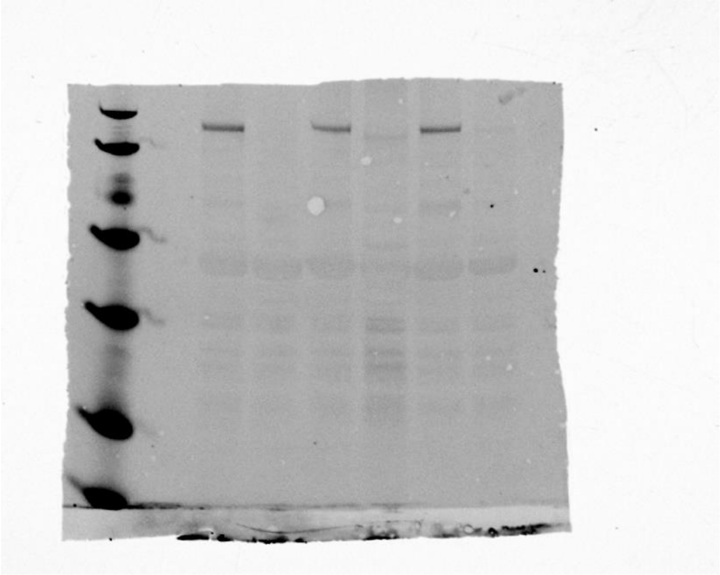

ImageJ Analysis

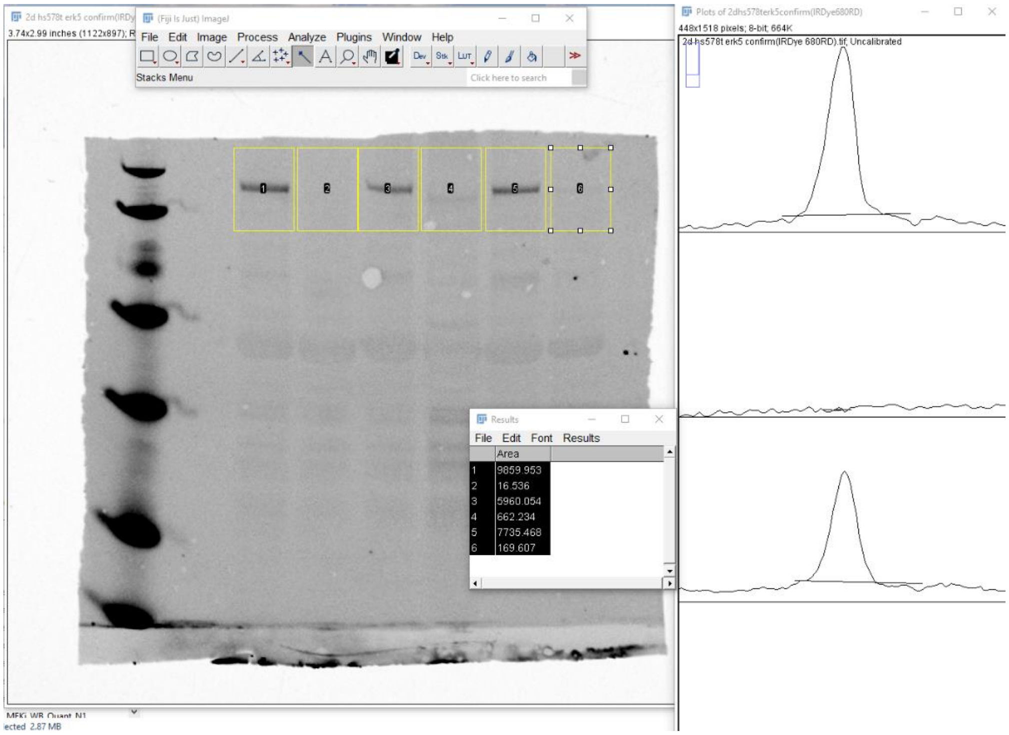

2. NF*κ*B/RelA – IRDye 800CW

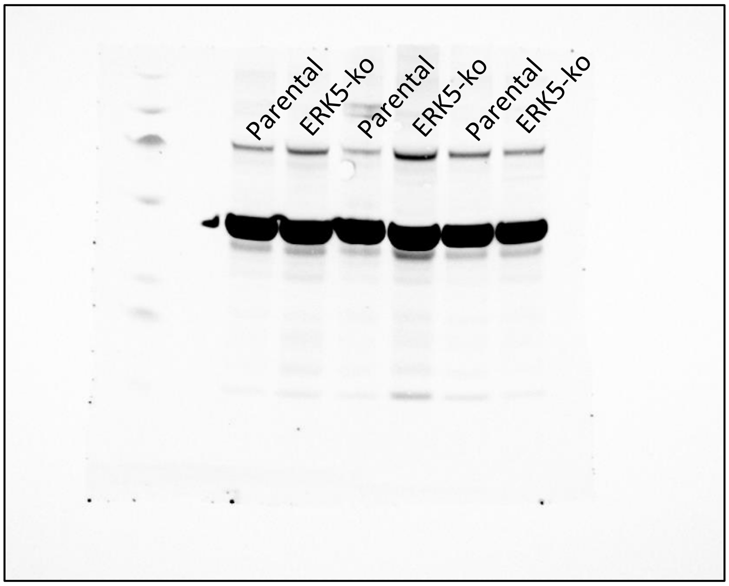

ImageJ Analysis

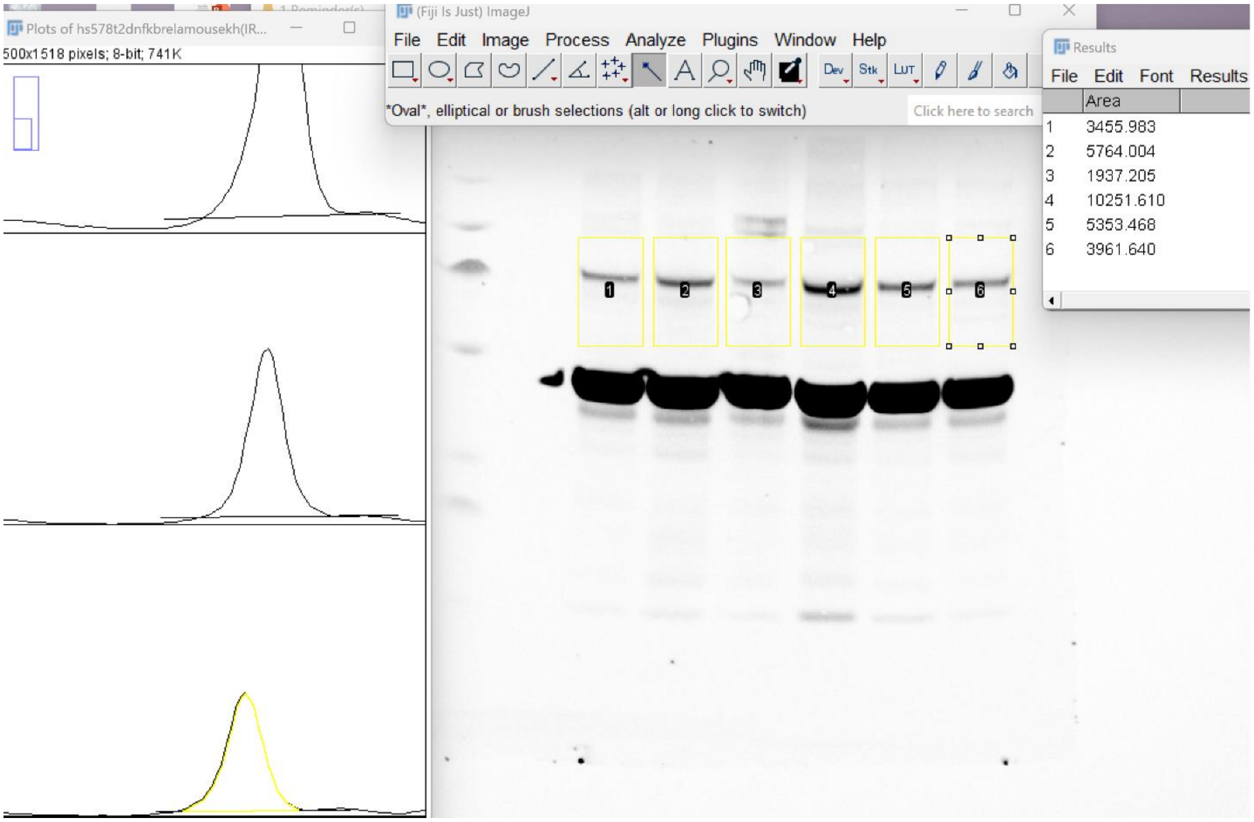

3. <u>pNFκB – IRDye 680RD</u>

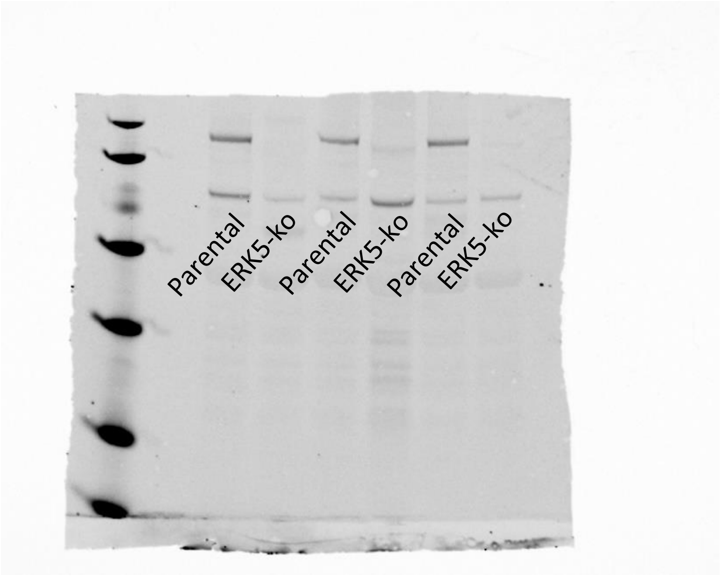

ImageJ Analysis

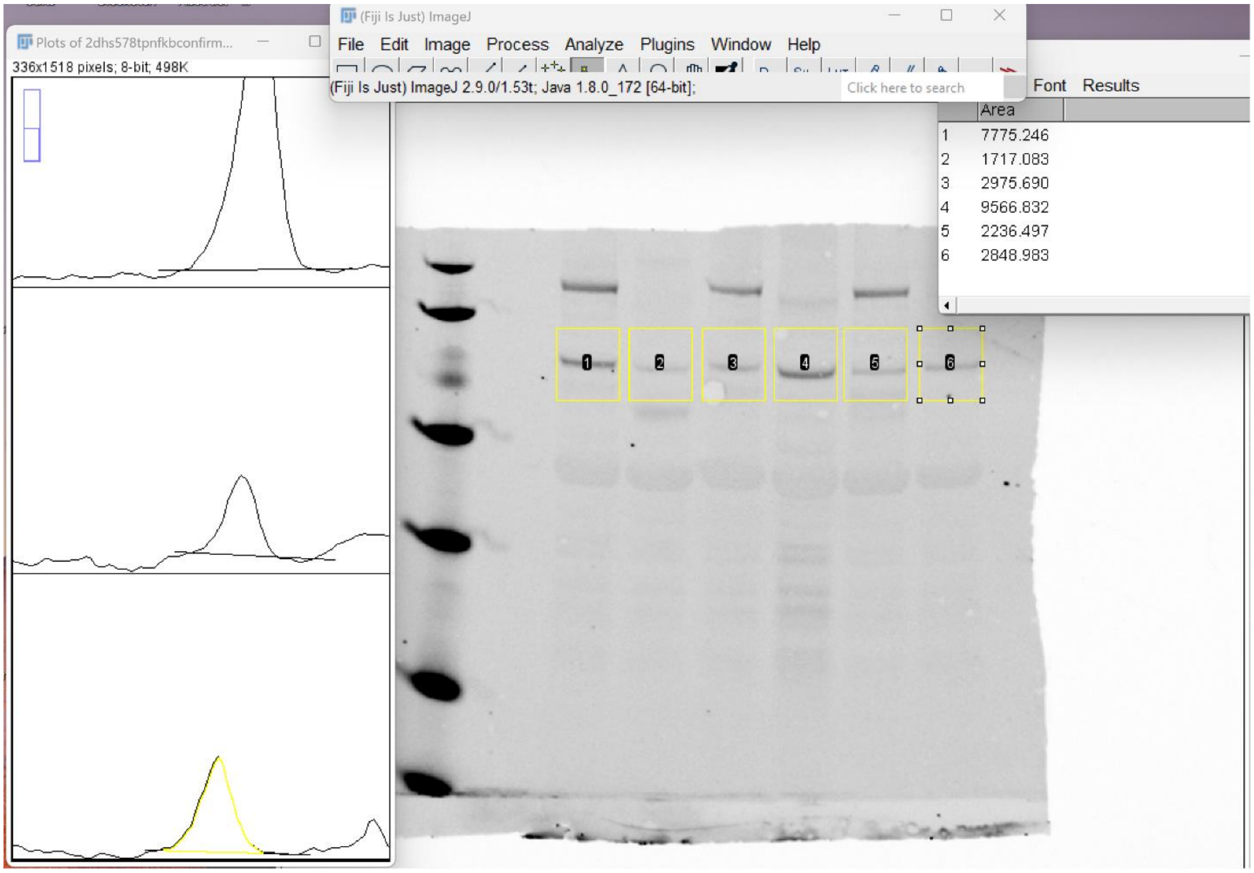

4. <u>NFκB2 (p100 and p52) – IRDye 680RD</u>

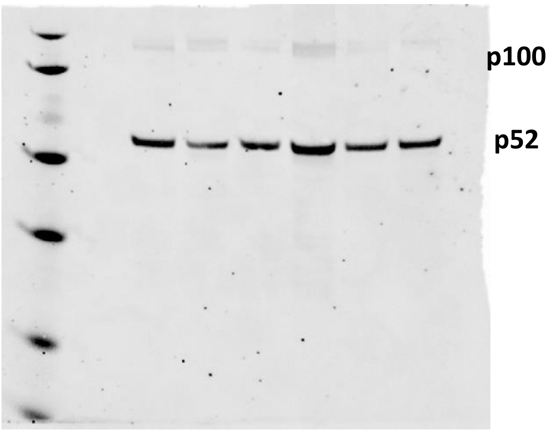

ImageJ Analysis

p52

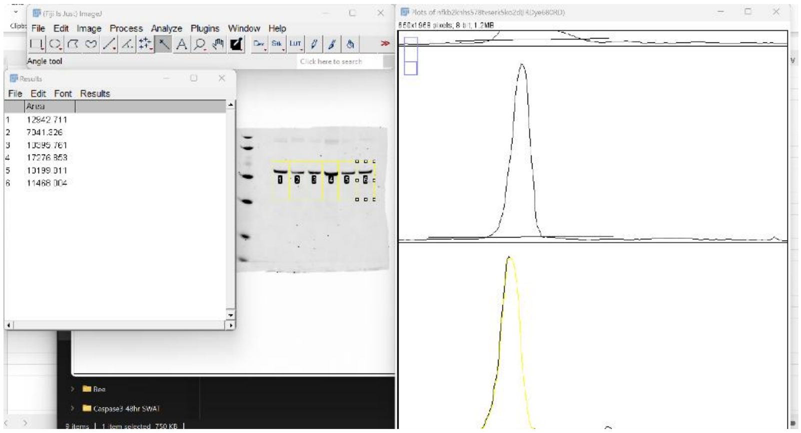

p100

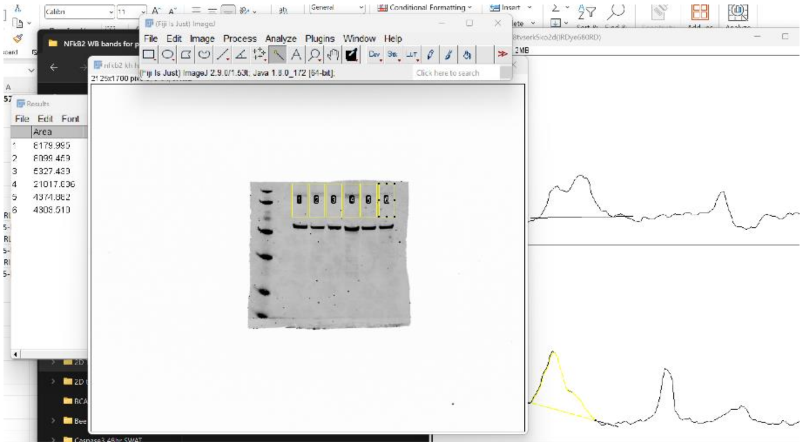

5. <u>β-Actin – IRDye 800CW</u>

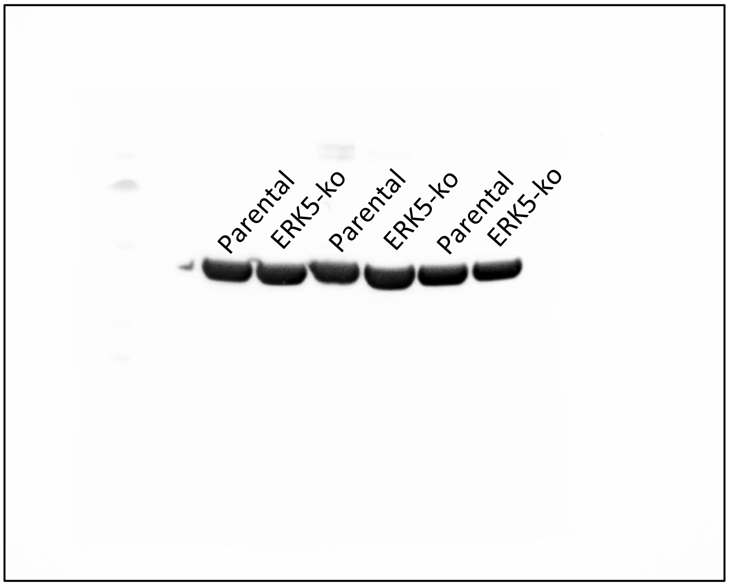

ImageJ Analysis

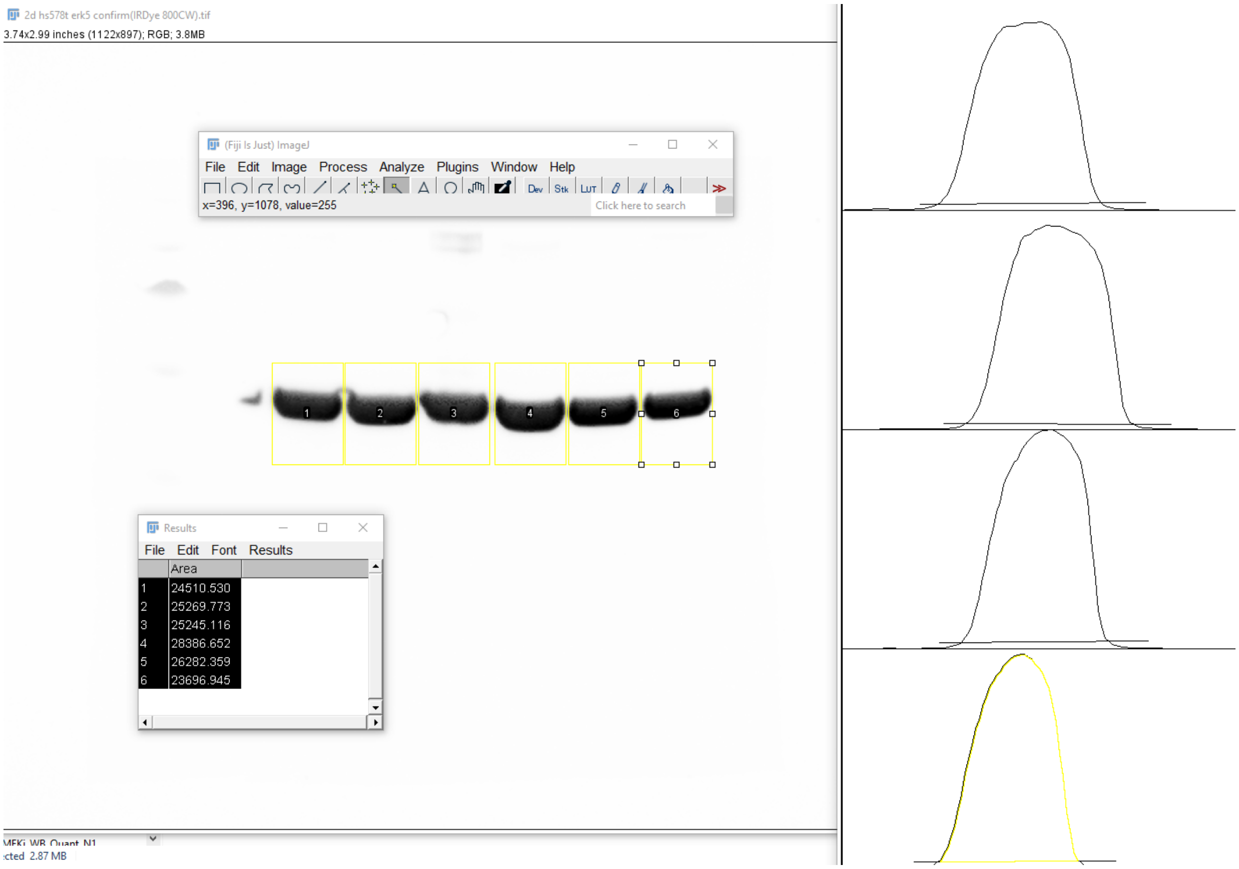

Supplemental Figure S8C1-5: Original western blots and ImageJ analysis of MDA-MB-231 parental and ERK5-ko cells cultured in 3D. Cropped western blots from 1-5 are shown in Figure 2C.

1. <u>IκBα – IRDye 800CW</u>

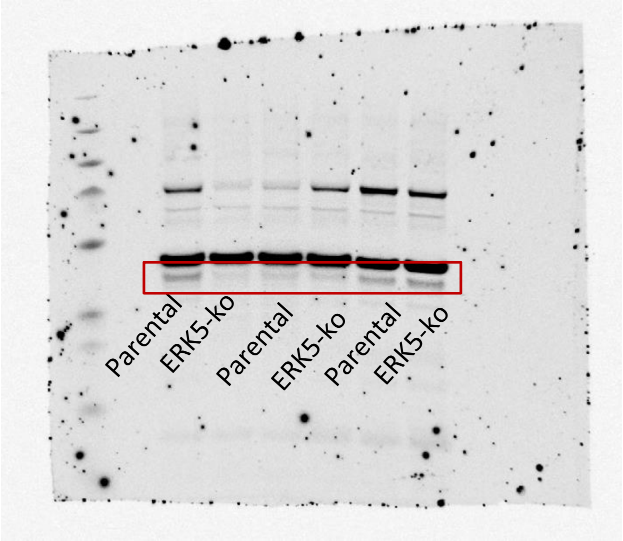

ImageJ analysis

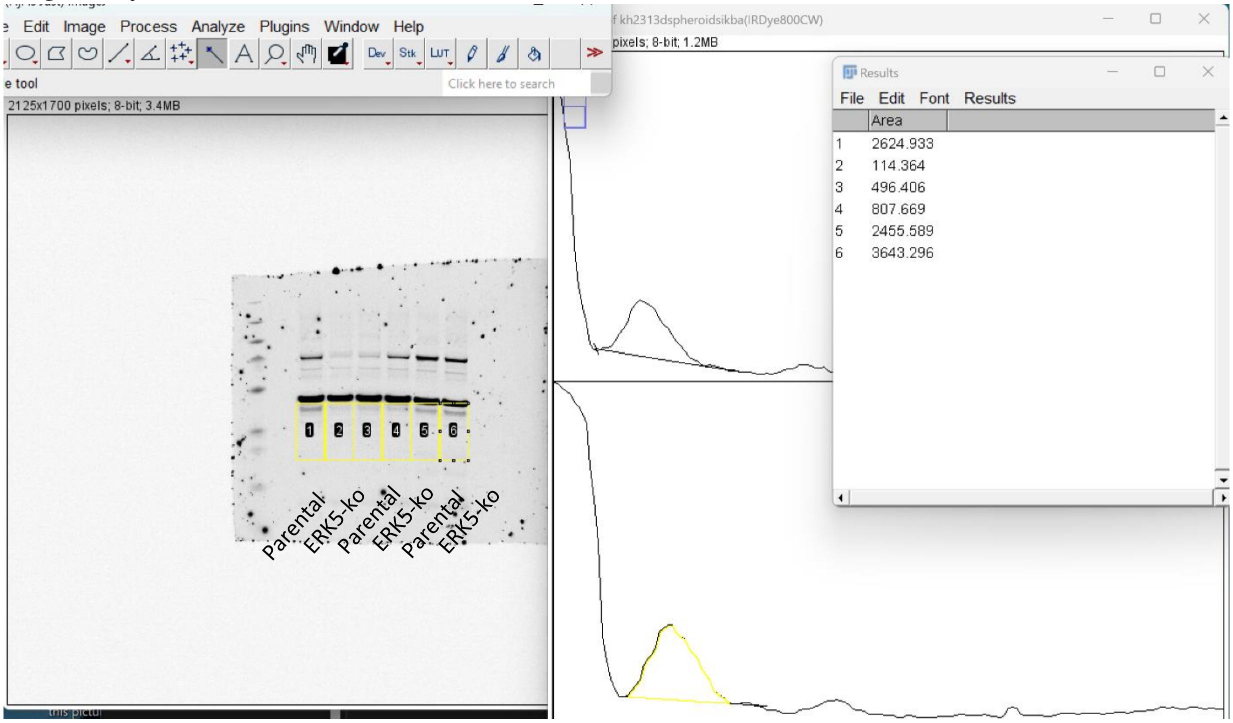

2. <u>NFκB/RelA – IRDye 800CW</u>

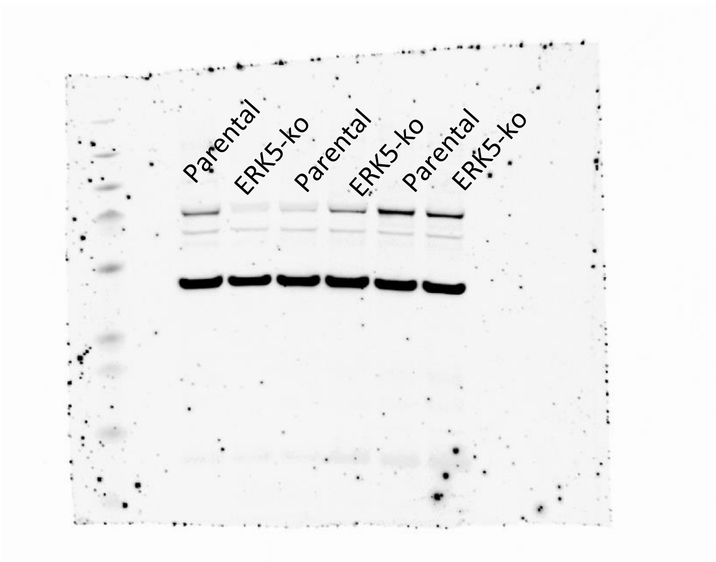

ImageJ Analysis

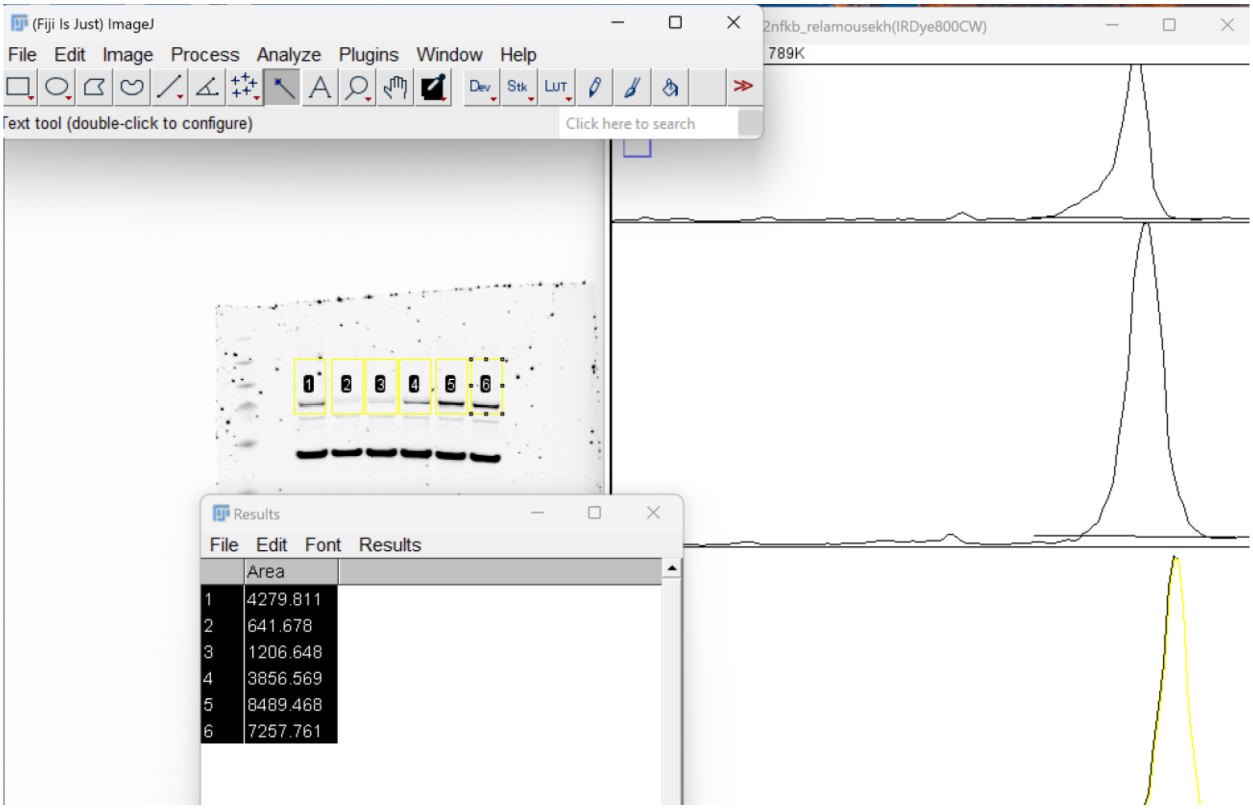

3. <u>pNFκB – IRDye 680RD</u>

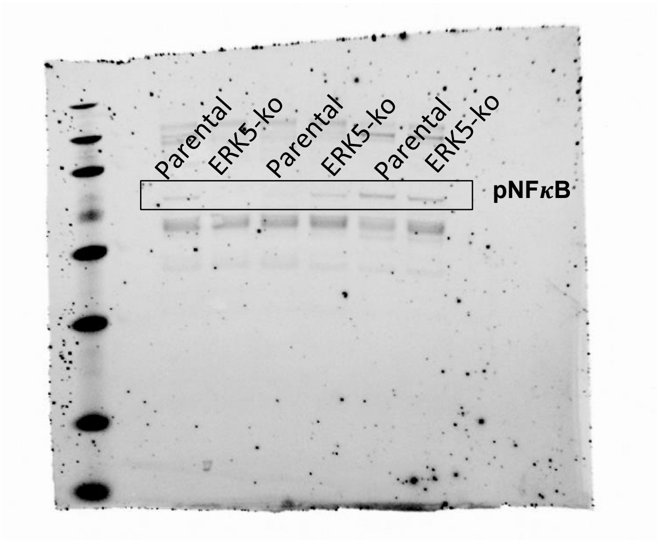

ImageJ Analysis

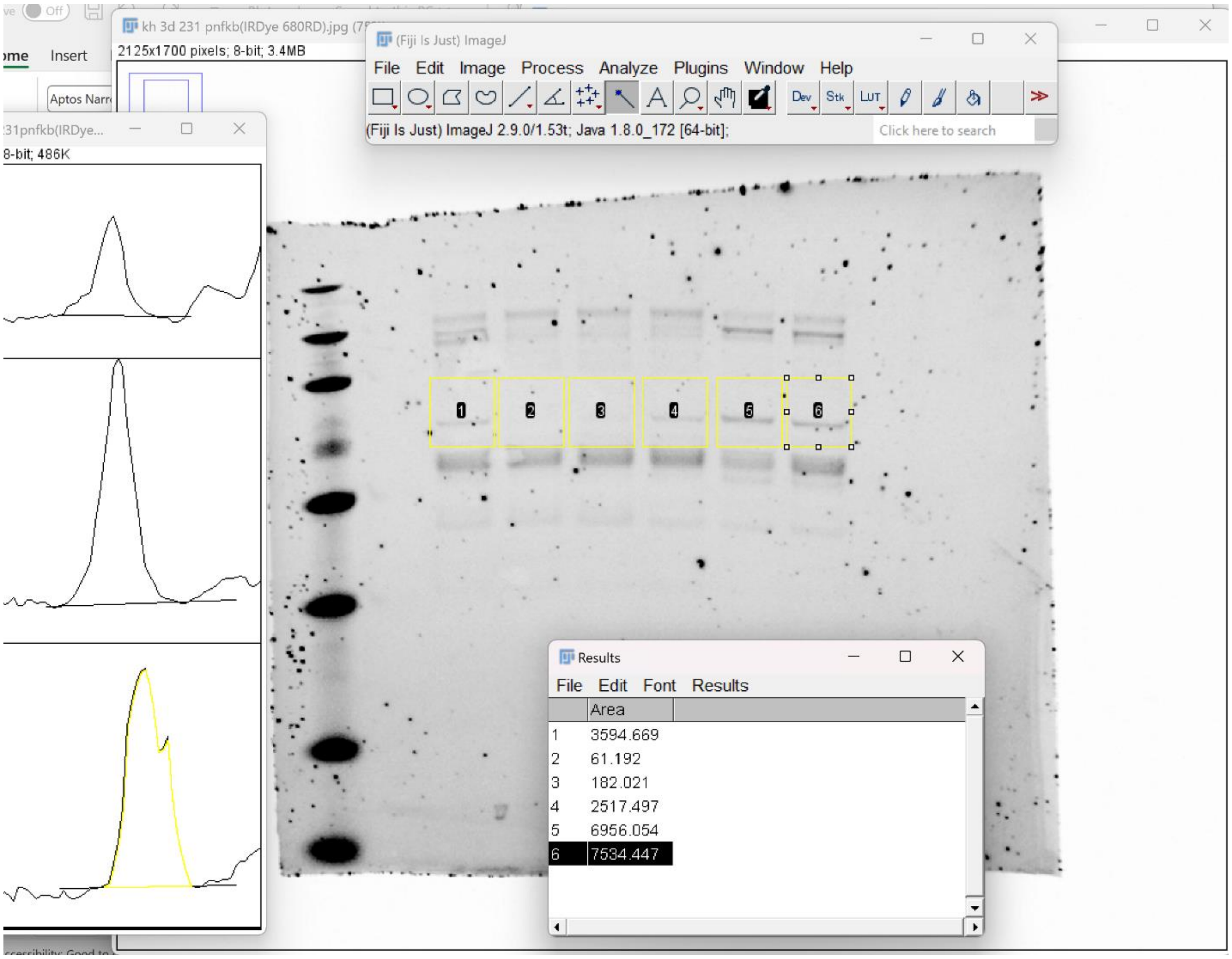

4. <u>NFκB2 (p100 and p52) – IRDye 680RD</u>

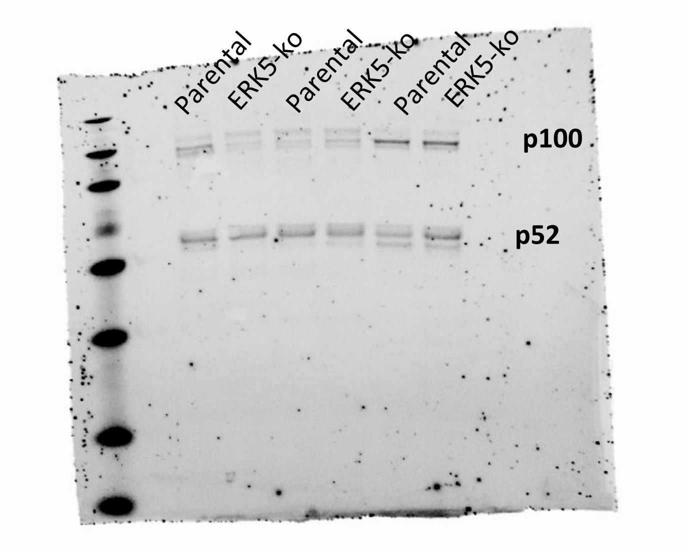

ImageJ Analysis - p100

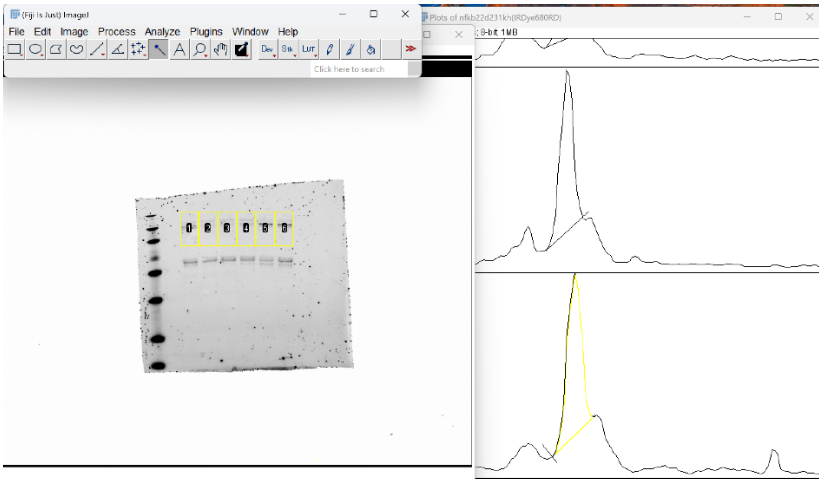

p52

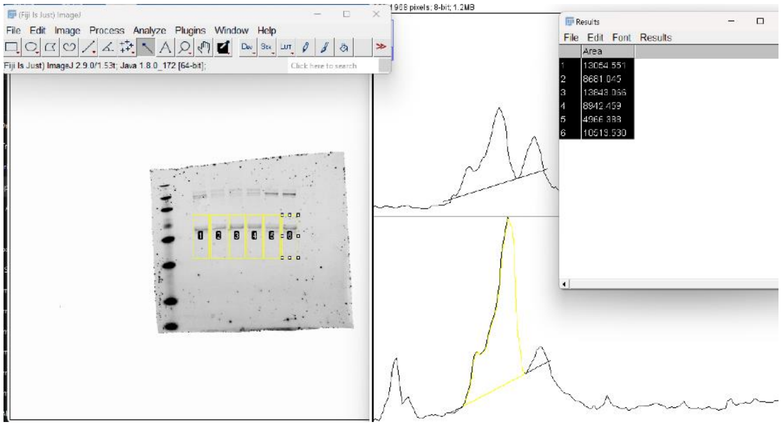

5. β-Actin – IRDye 800CW

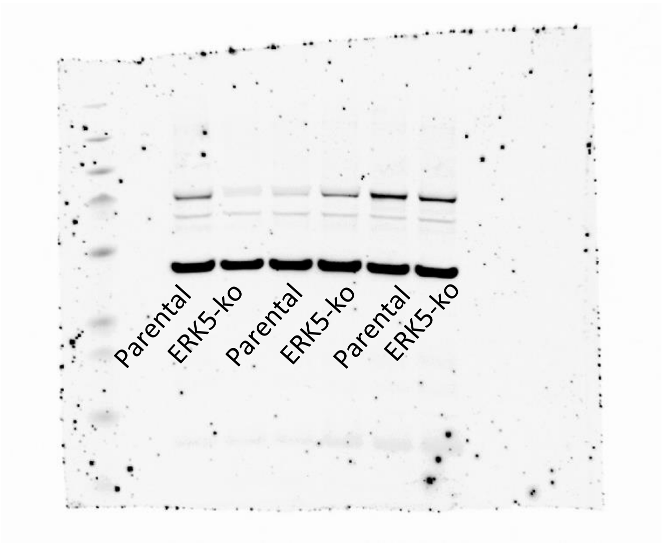

ImageJ Analysis

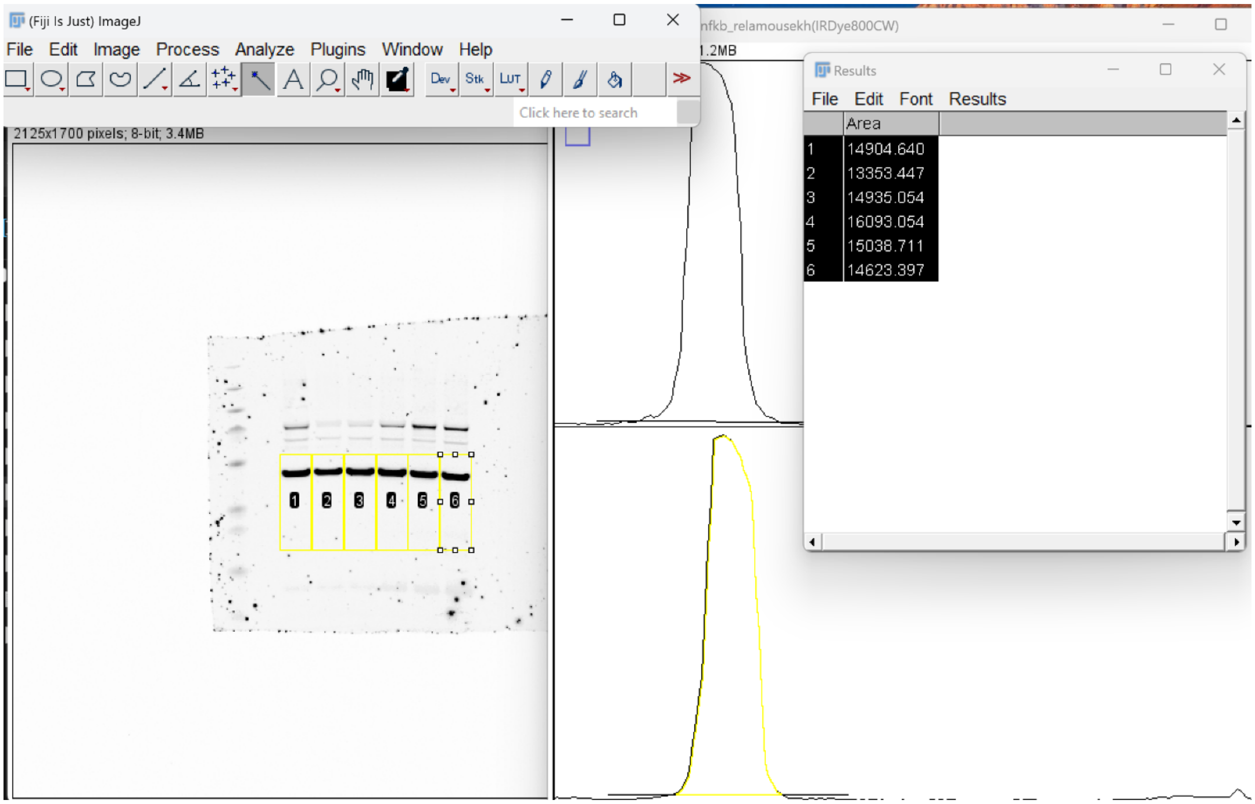

Supplemental Figure S8D1-5: Original western blots and ImageJ analysis of Hs578T parental and ERK5-ko cells cultured in 3D. Cropped western blots from 1-5 are shown in Supplemental Figure 4B.

1. <u>IκBα – IRDye 800CW</u>

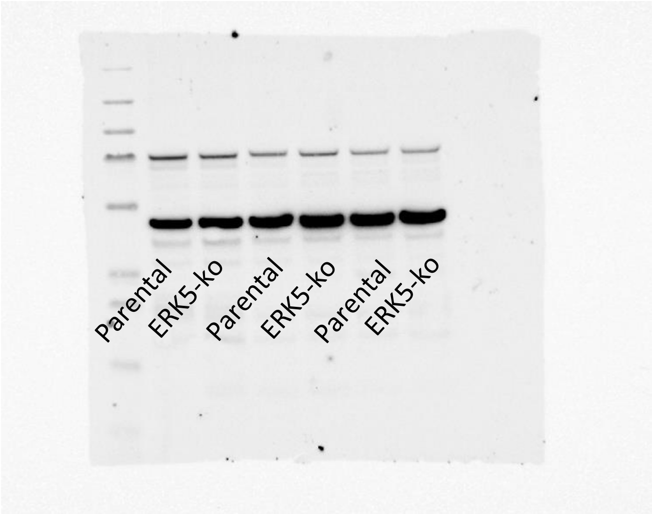

ImageJ Analysis

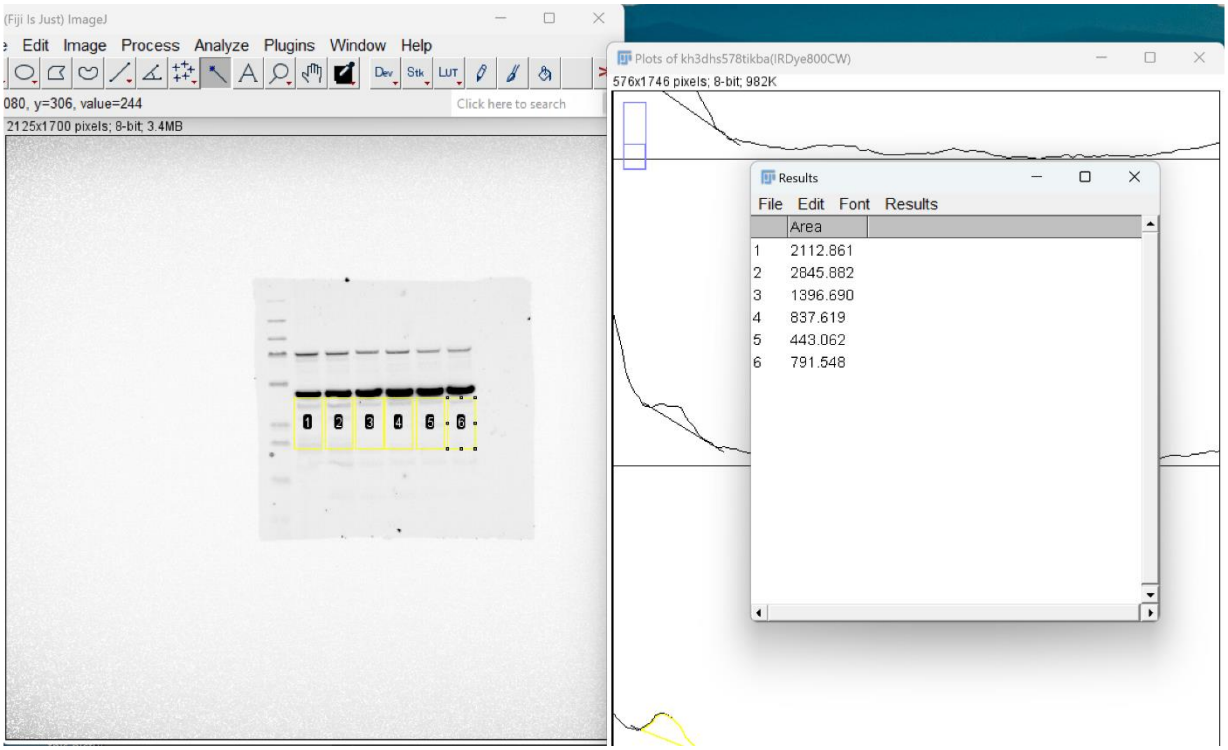

2. <u>NFκB/RelA – IRDye 800CW</u>

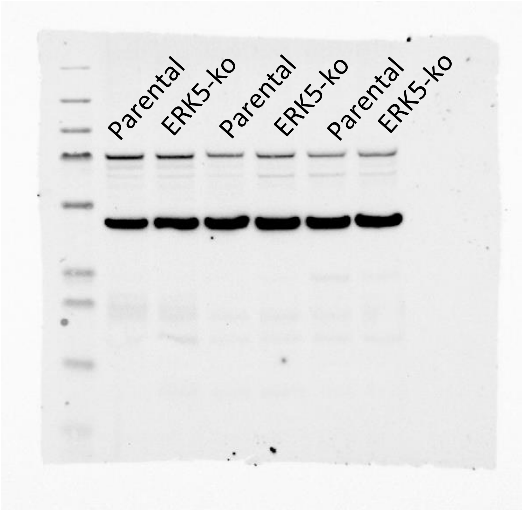

ImageJ Analysis

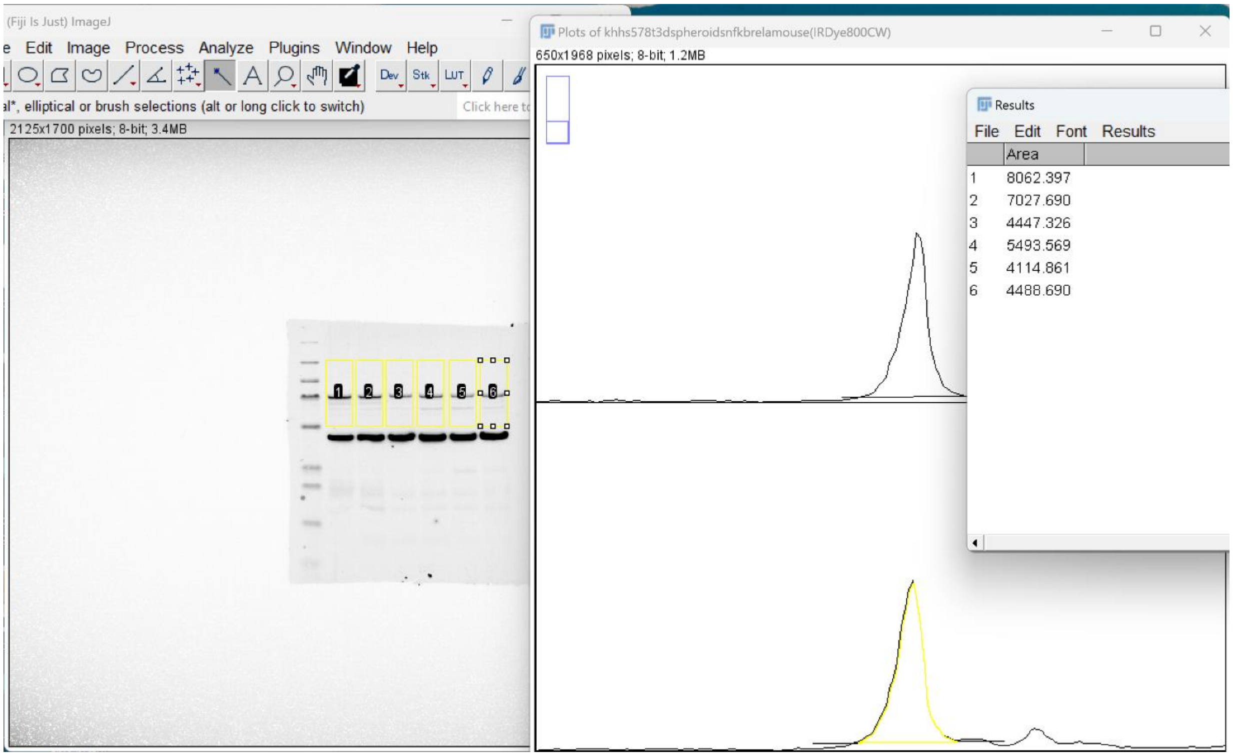

3. <u>pNFκB – IRDye 680RD</u>

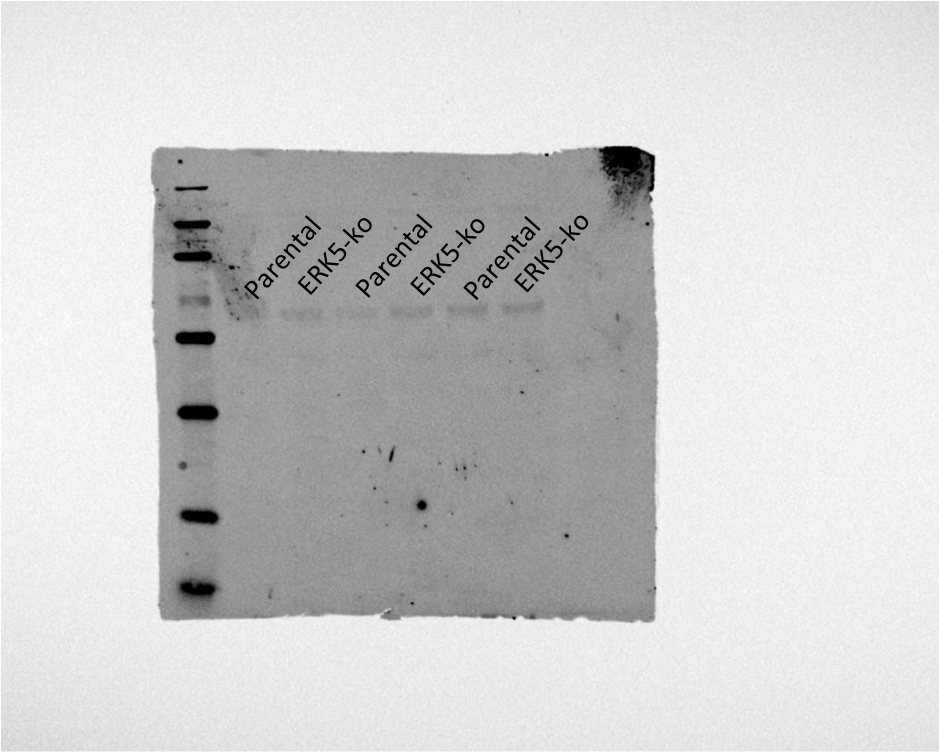

ImageJ Analysis

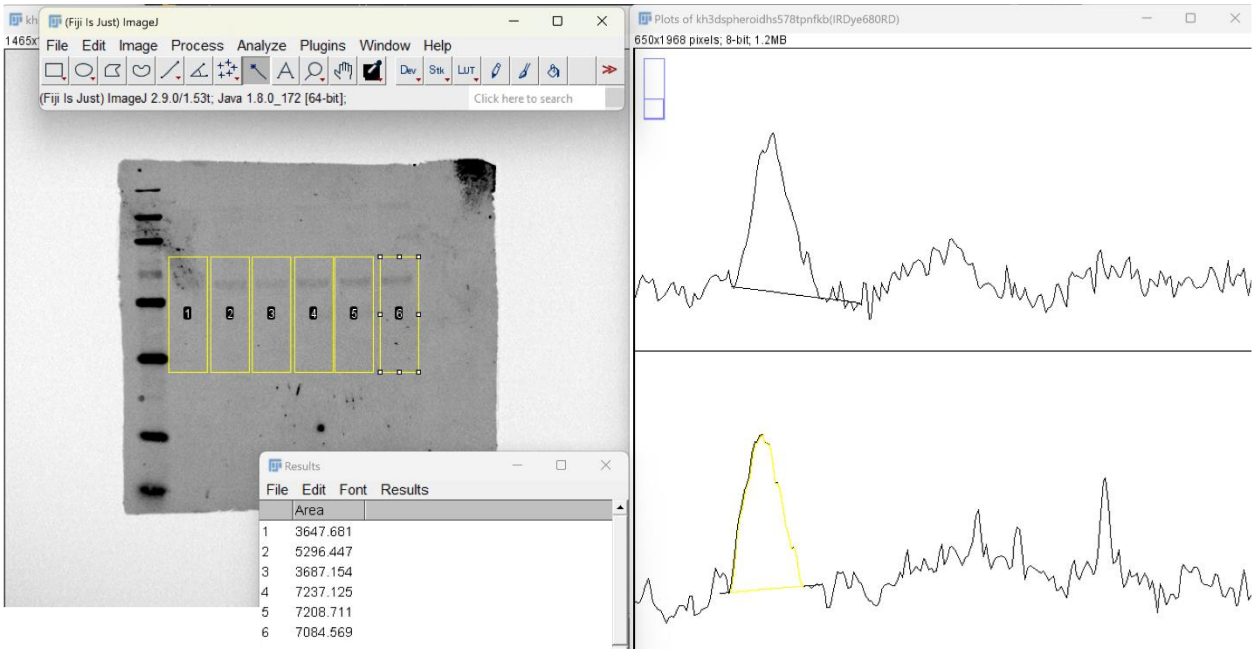

4. <u>NFκB2 (p100 and p52) – IRDye 680RD</u>

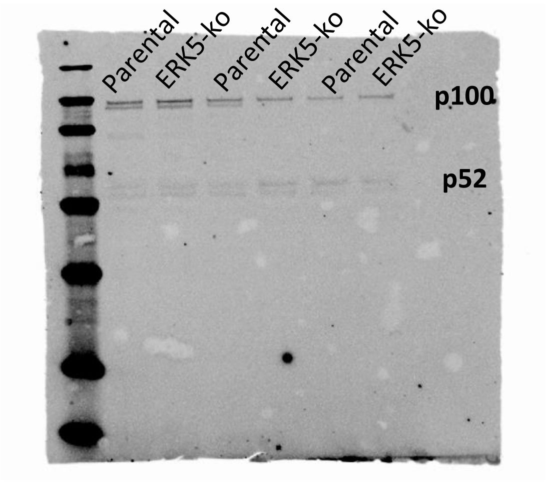

ImageJ Analysis

p52

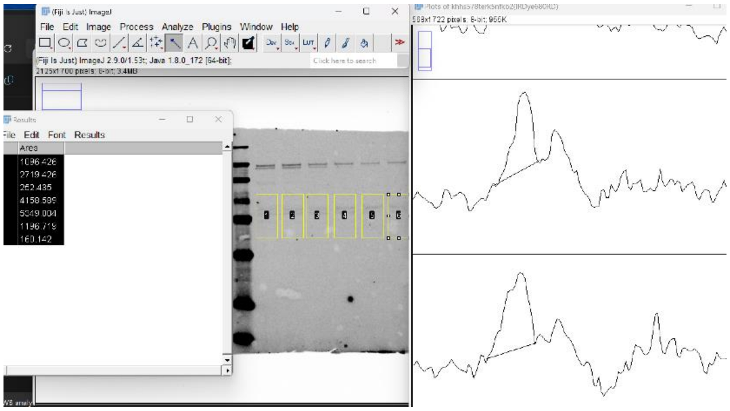

p100

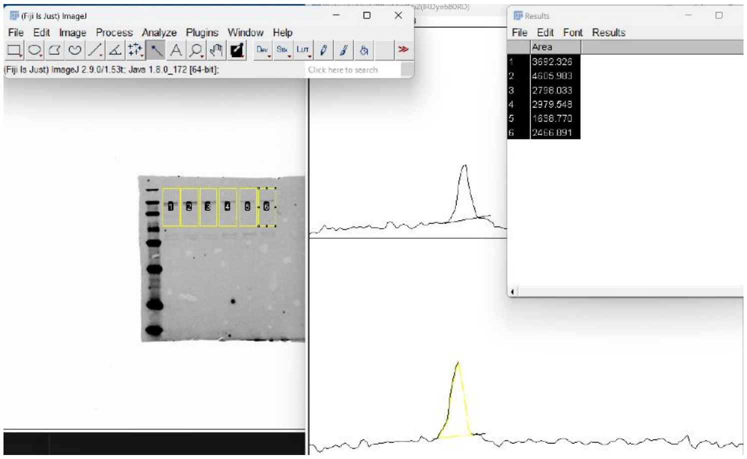

5. <u>β-Actin – IRDye 800CW</u>

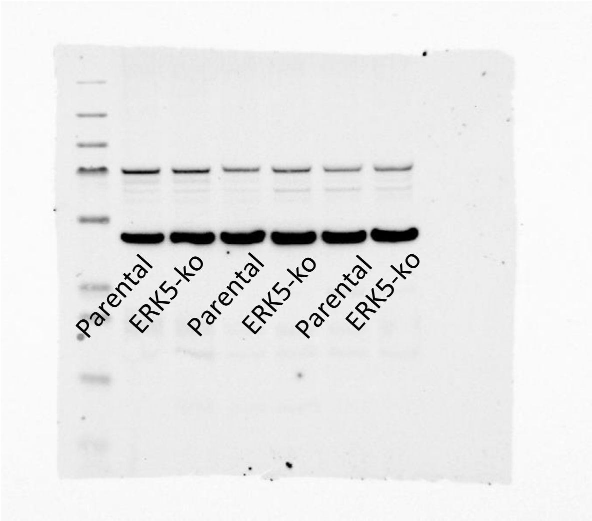

ImageJ Analysis

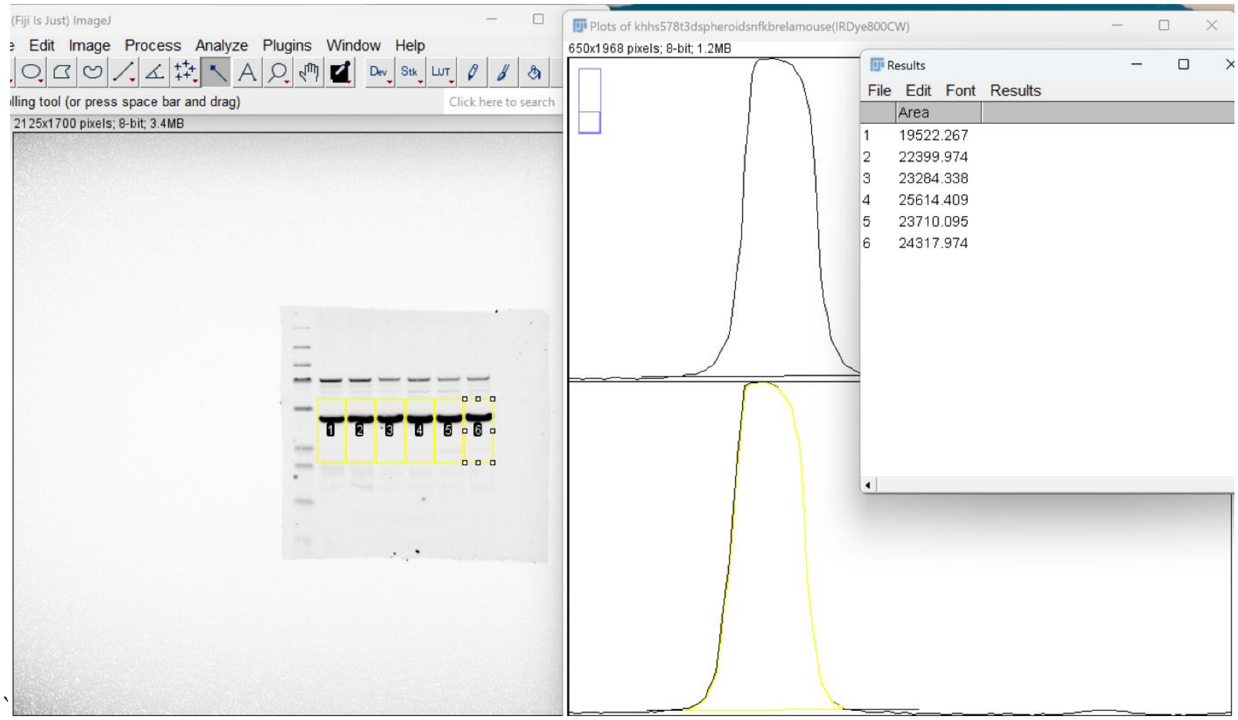

Supplemental Figure S8E1-4: Original western blots and ImageJ analysis of MDA-MB-231 parental and ERK5-ko cells cultured in BA-MaPS. Cropped western blots from 1-4 are shown in Figure 2D.

1. <u>NFκB/RelA – IRDye 800CW</u>

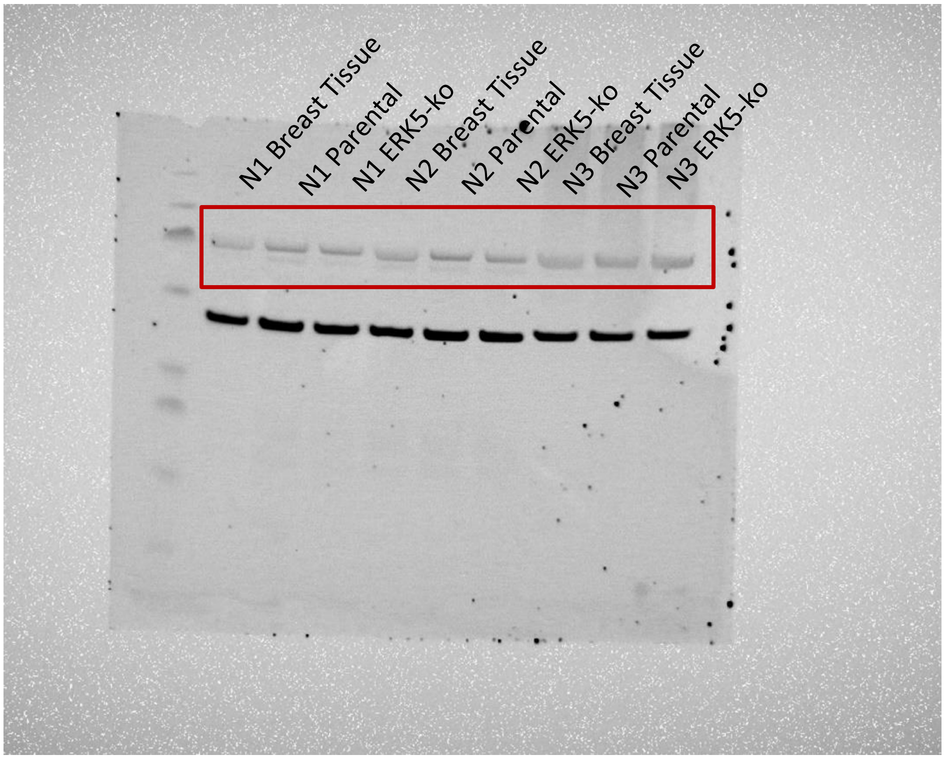

ImageJ Analysis

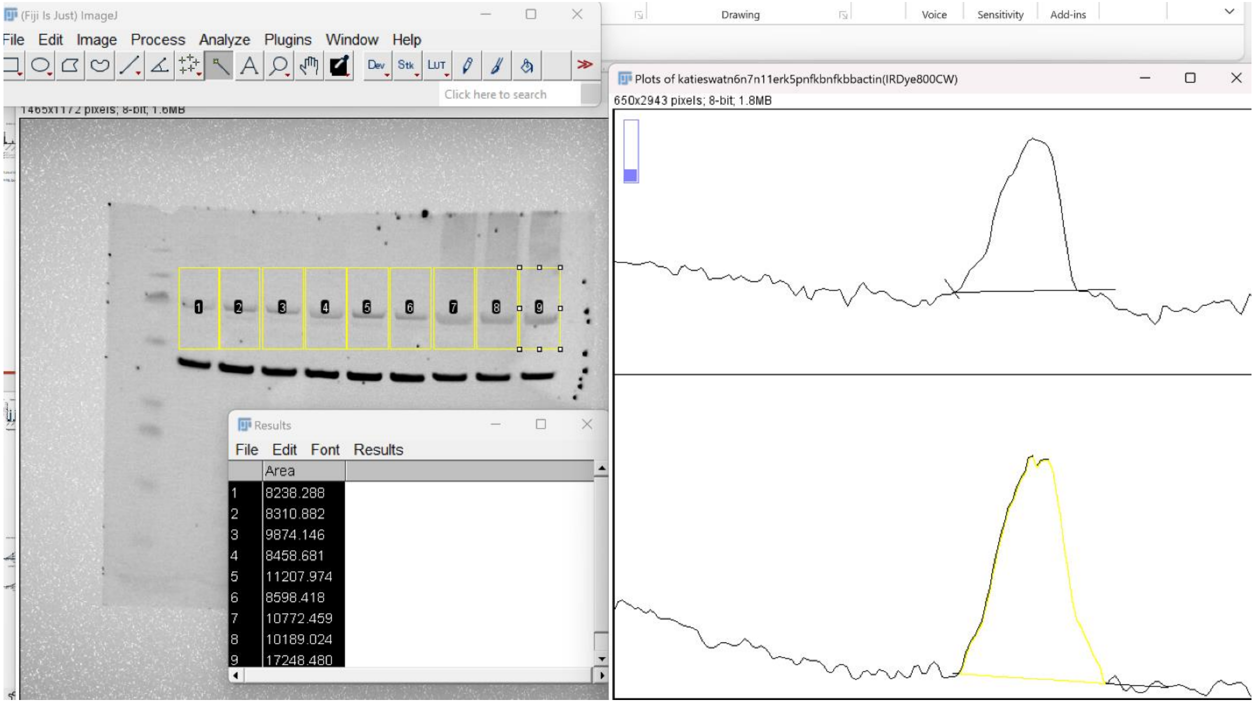

2. <u>pNFκB – IRDye 680RD</u>

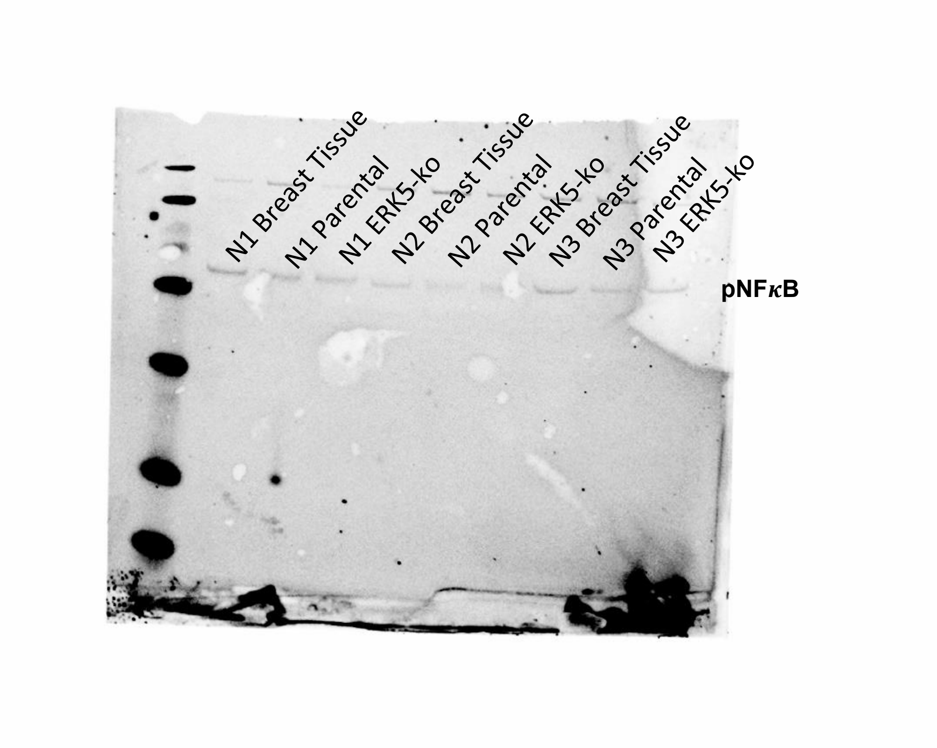

ImageJ Analysis

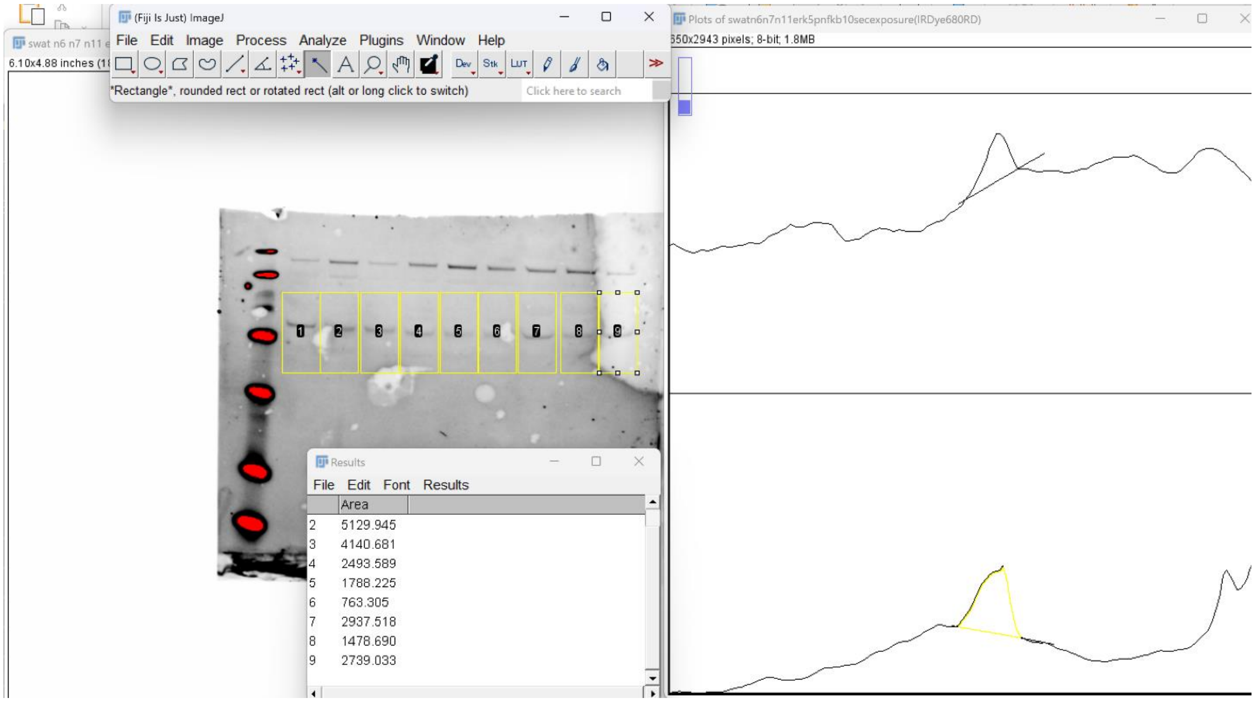

3. <u>ERK5 – IRDye 680RD</u>

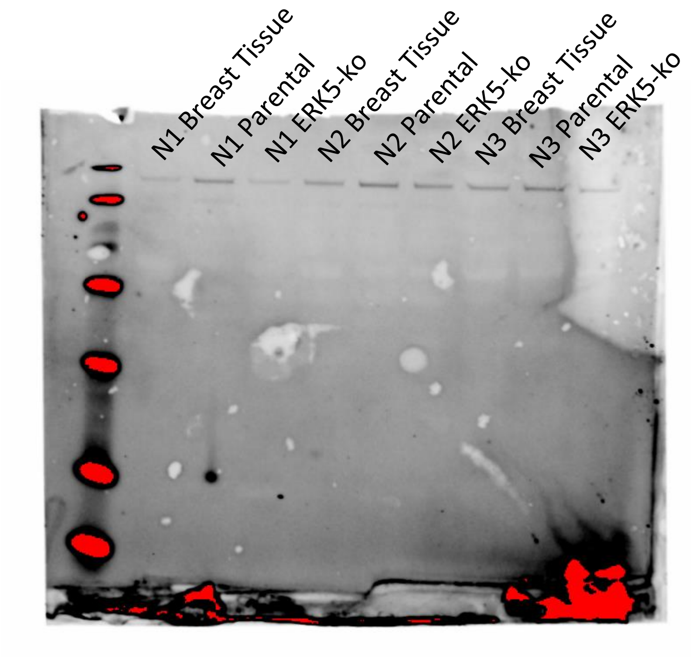

ImageJ Analysis

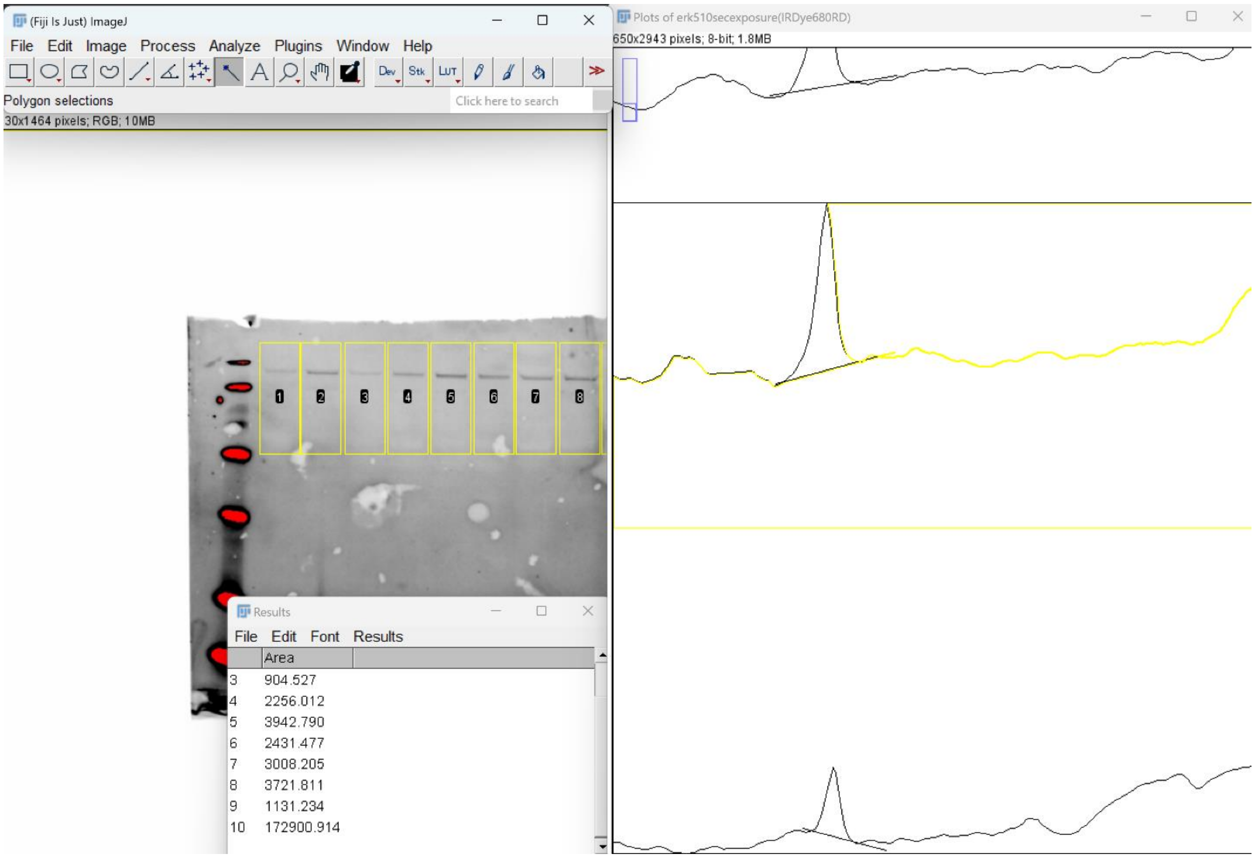

4. β-<u>Actin – IRDye 800CW</u>

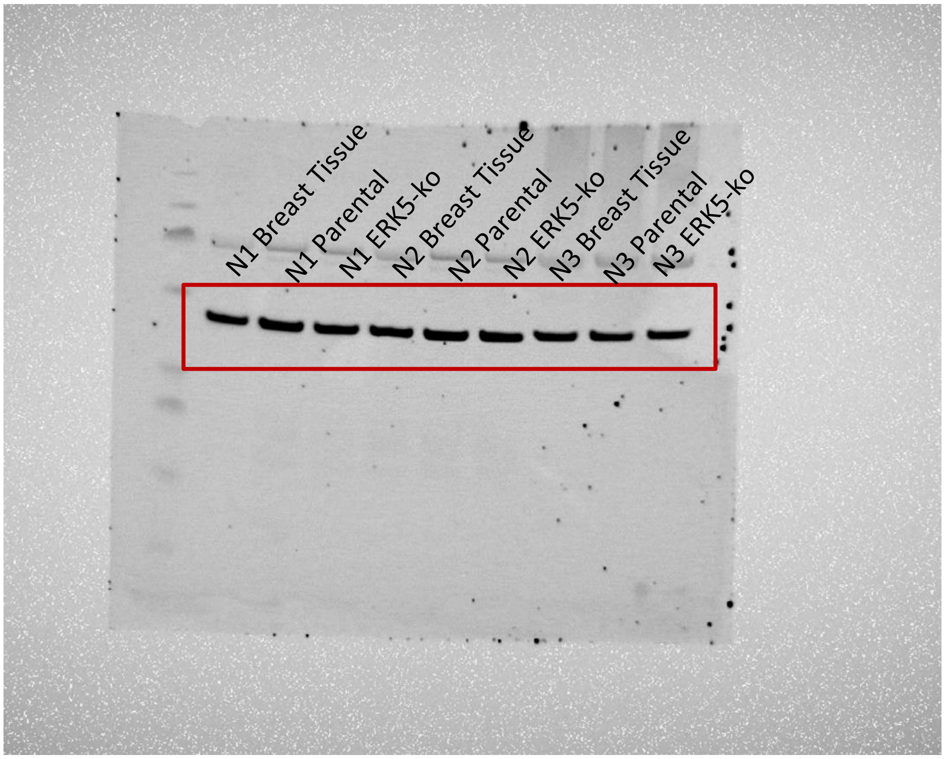

ImageJ Analysis

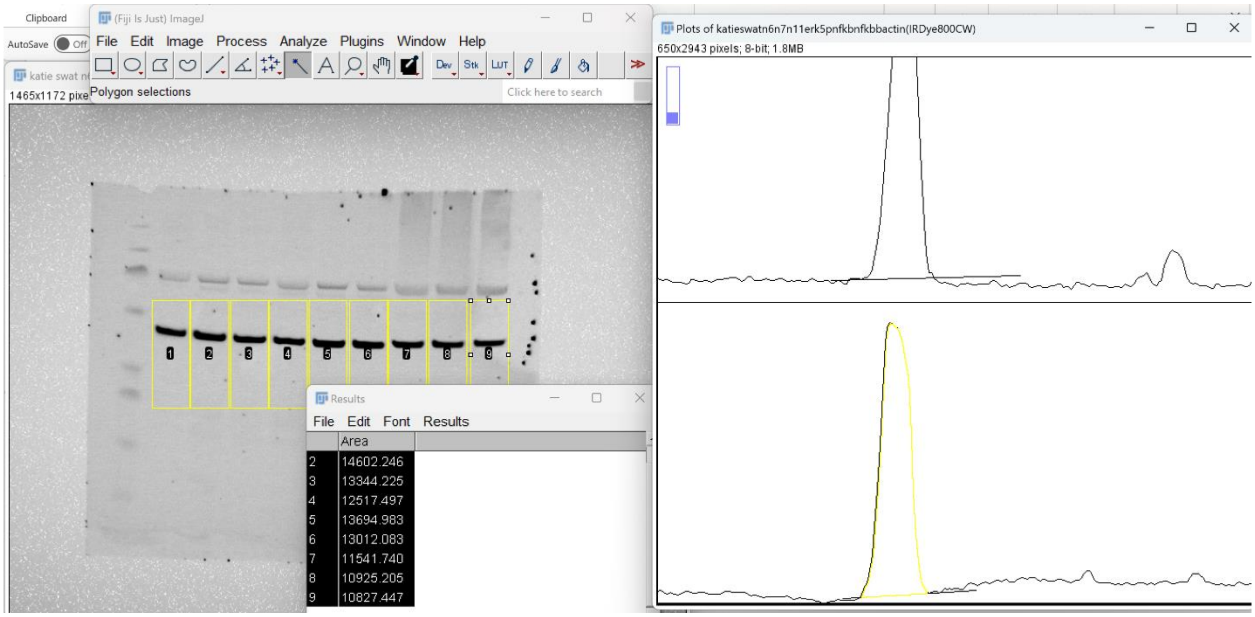

Supplement: Supplementary file 1 [file cancers-18-00376-s001.zip › Supplemental Figure S8A-E_OrigionalWesternBlotImages.pdf]
