# Supplementary material for: Dissecting Context-Specific Effects of ERK5 Signaling in Triple-Negative Breast Cancer
Source: Cancers (Basel). 2026 Jan 26;18(3):376. doi: 10.3390/cancers18030376 (PMC12896816; doi:10.3390/cancers18030376)
Supplement: Supplementary file 1 [file cancers-18-00376-s001.zip › Supplemental Figures S1-S7.pdf]

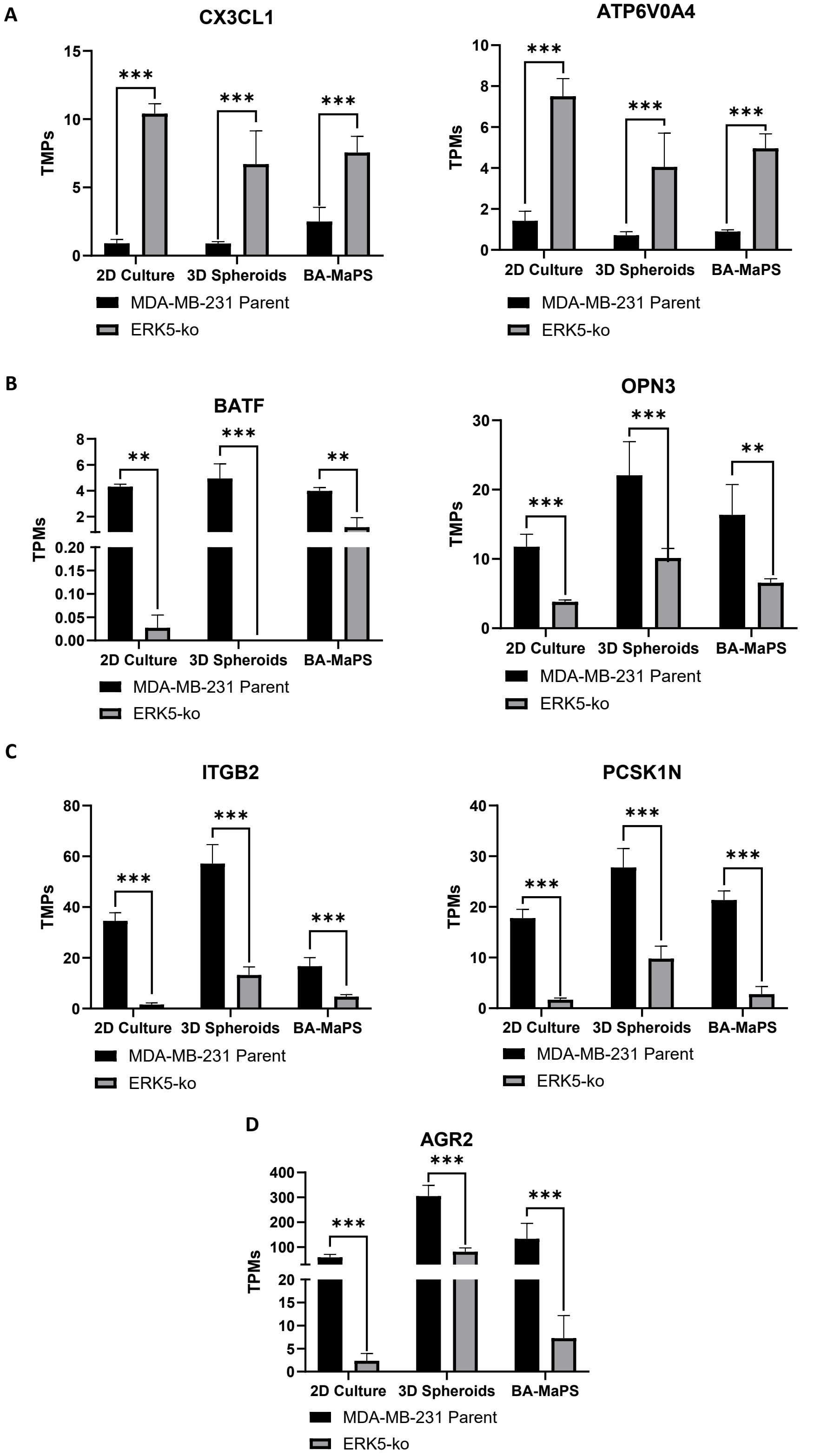

Supplemental Figure S1: Overlapping upregulated and downregulated transcript in 2D culture, 3D spheroid, and BA-MaPS. A) Transcripts per million (TPMs) of the genes upregulated in apical surface proteins pathway. **B)** TPMs of the genes downregulated in estrogen response late pathway. **C)** TPMs of the genes downregulated in Kras signaling up pathway. **D)** TPMs of gene downregulated in both estrogen response late and Kras signaling up pathways.

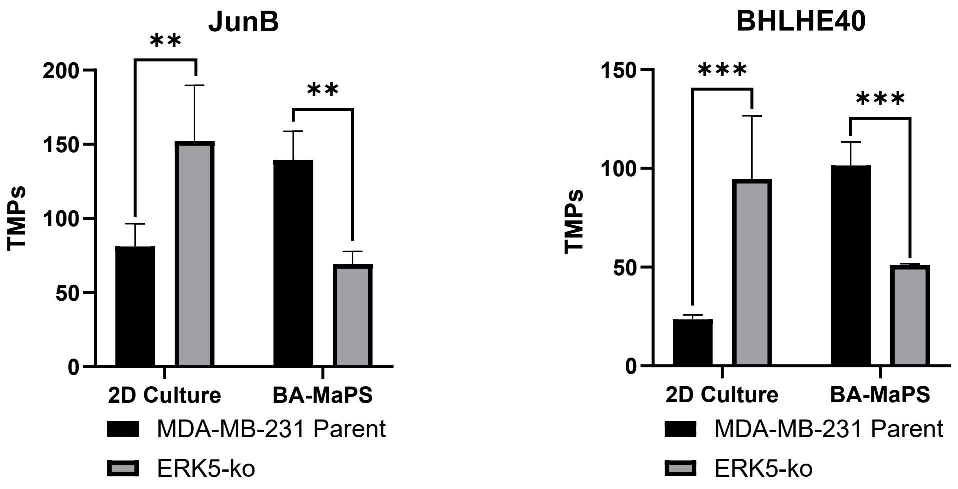

Supplemental Figure S2: Overlapping modified genes in the NFκB pathway in 2D and BA-MaPS. Transcripts per million (TPMs) of the genes JunB, and BHLHE40.

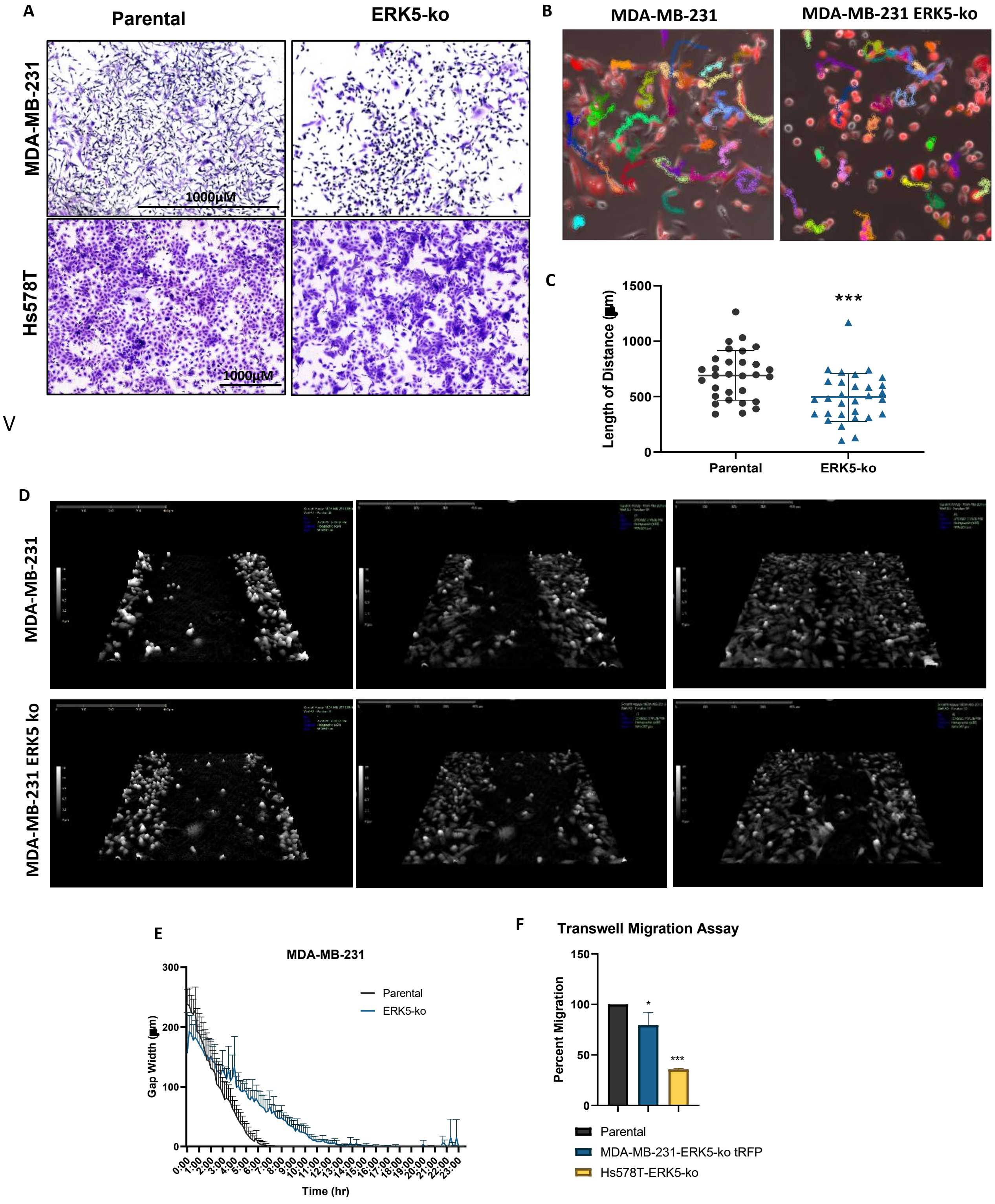

Supplemental Figure S3: ERK5 migration analysis confirmation in 2D culture. Previously, our lab showed ERK5 deletion changes phenotype and decreases migration in 2D culture. We further confirmed these results in two TNBC cell lines, MDA-MB-231 and Hs578T through **A)** crystal violet images, **B,C)** time-lapse imaging, **D,E)** scratch/wound healing assay, and **F)** transwell migration assay. * $p \leq 0.05$, ** $p \leq 0.01$, *** $p \leq 0.001$.

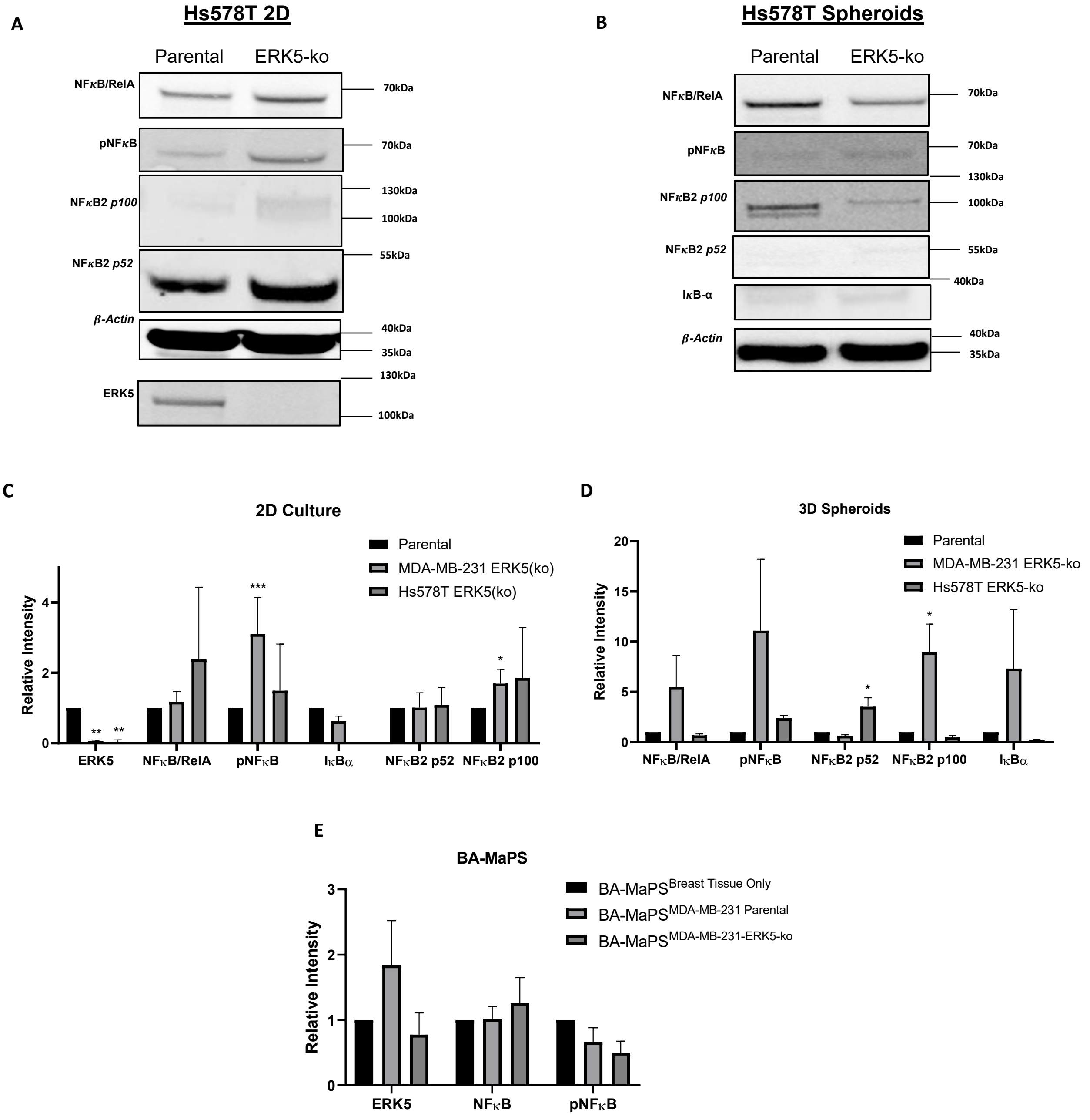

Supplemental Figure S4: Western blot analysis of NFκB pathway members in MDA-MB-231 and Hs578T. A) NFκB protein expression in Hs578T parental and ERK5-ko cells after 2D culture, and **B)** 3D spheroids culture. **C)** The relative protein intensity of cells cultured in 2D, **D)** 3D spheroids, and **E)** BA-MaPS was normalized to β-actin and parental and samples were run in triplicate experiments ± SEM. * $p \leq 0.05$, ** $p \leq 0.01$, *** $p \leq 0.001$.

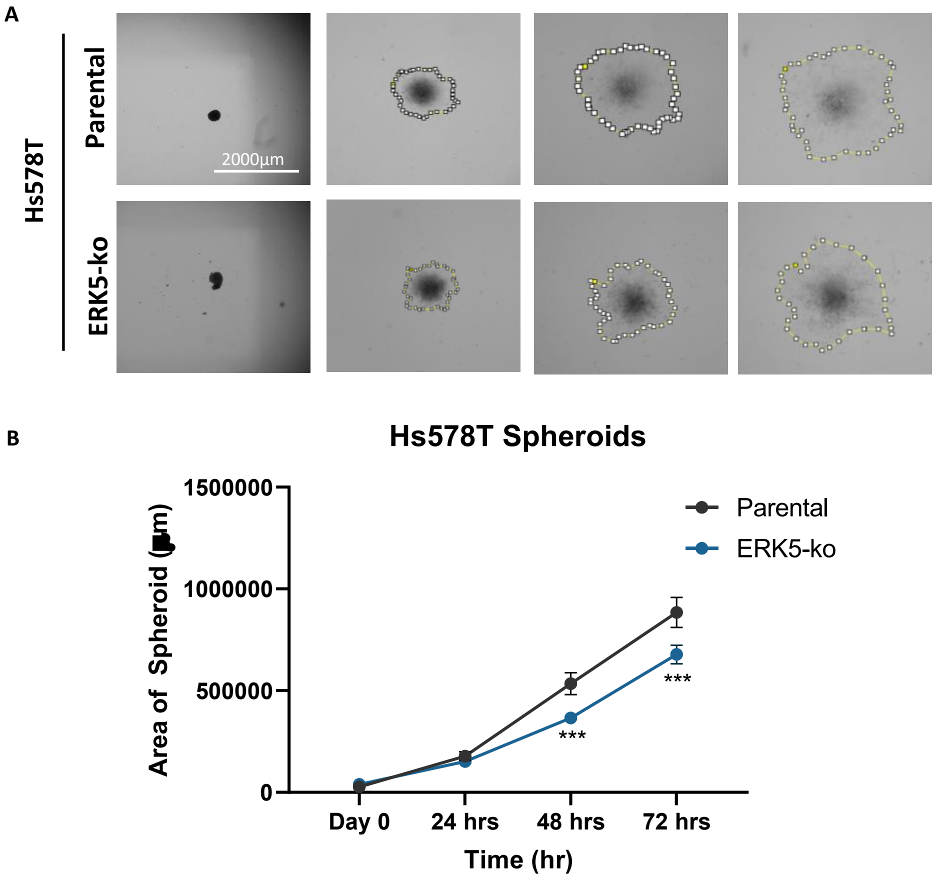

Supplemental Figure S5: Hs578T 3D spheroid pseudo-migration assay. A) Representative images of MDA-MB-231 parental and ERK5-ko spheroid pseudo-migration assay over 72hrs. **B)** The area of spheroid was quantified after 24 hrs, 48hrs, and 72hrs in Fiji/ImageJ and normalized to Day 0. Experiments were completed in triplicate ± SEM. *** $p \leq 0.001$

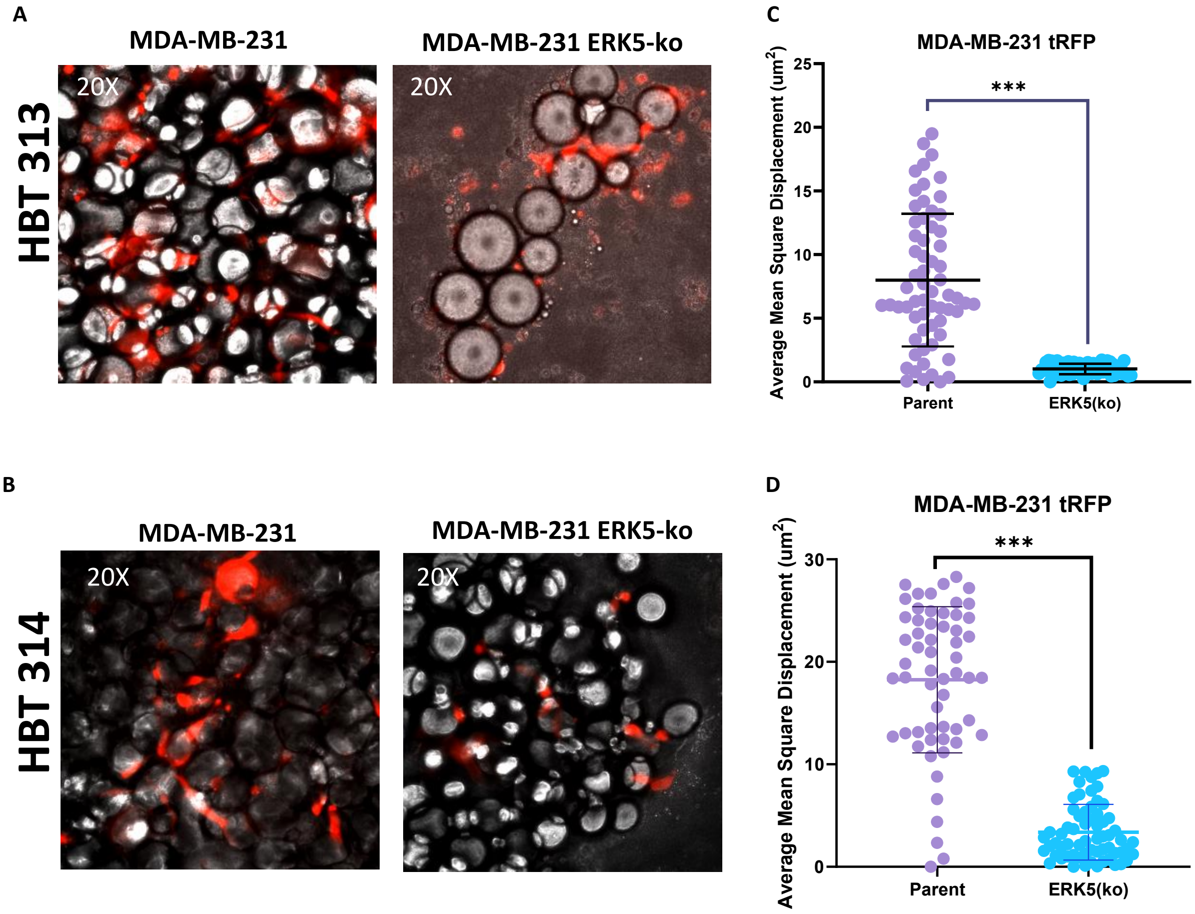

Supplemental Figure S6: Time-lapse migration assay in MDA-MB-231 parental and ERK5-ko cells seeded in BA-MaPS with additional donors outlined in Figure 5. Representative videos show time-lapse imaging was used to record cell movement and activity of MDA-MB-231-ERK5-ko and parental lines in BA-MaPS on two additional breast doners, **A)** HBT313 and **B)** HBT314. **C)** Cell tracking analysis was completed on the ImageJ add-ons MTrack and ADAPT for HBT313 and **D)** HBT314, *** $p \leq 0.001$

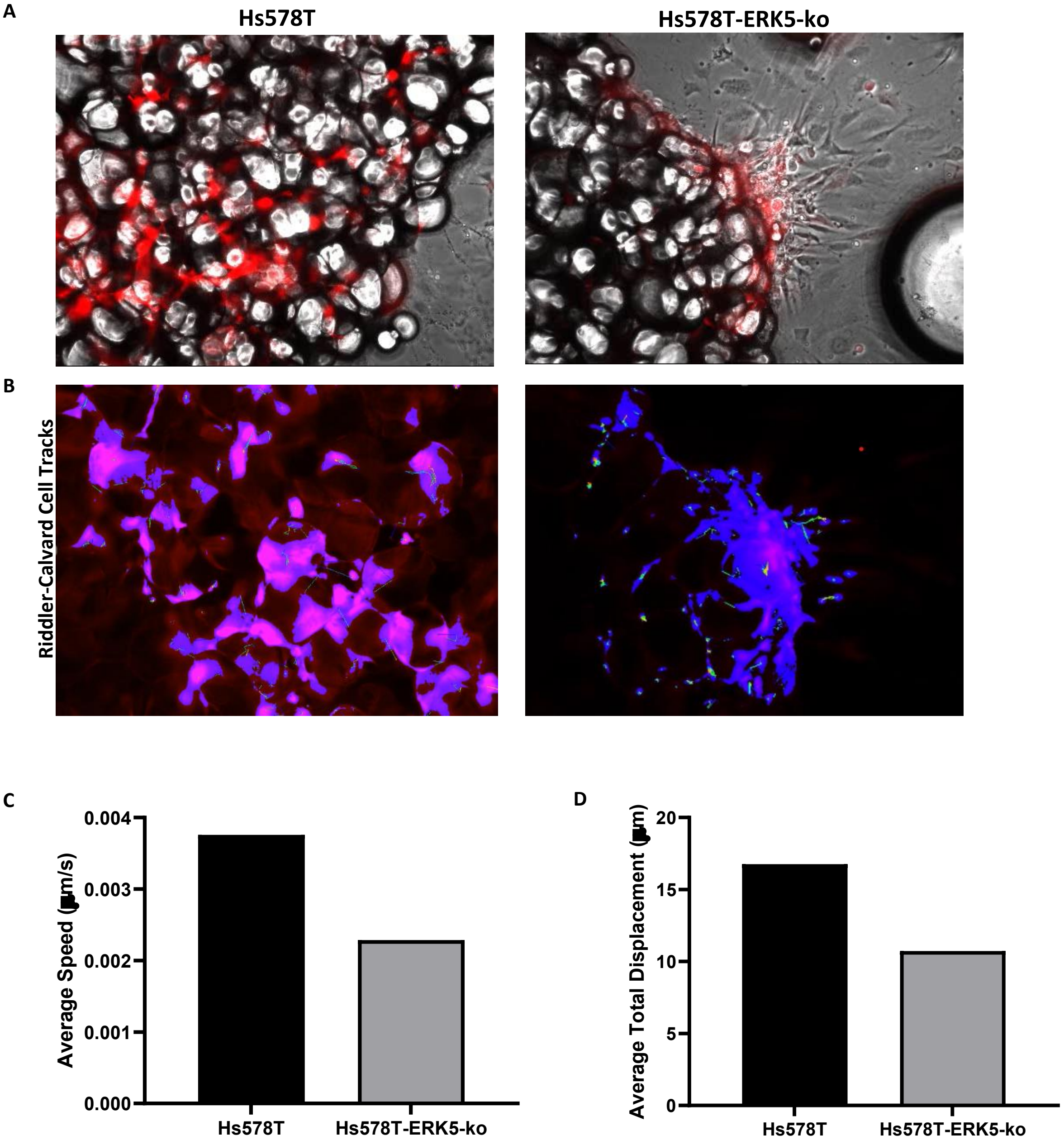

Supplemental Figure S7: Time-lapse migration assay in Hs578T parental and ERK5-ko cells seeded in BA-MaPS. A) Representative videos show time-lapse imaging was used to record cell movement and activity of Hs578T-ERK5-ko and the parental line in BA-MaPS in one female breast doner (Race: Black; BMI: 38.4; Age: 42). Images were taken every 30 mins. for 48 hrs. **B)** Riddler-Calvard masking was used to analyze cell tracks and determine the **C)** average speed and **D)** average total displacement.
