# Supplementary material for: Dissecting Context-Specific Effects of ERK5 Signaling in Triple-Negative Breast Cancer
Source: Cancers (Basel). 2026 Jan 26;18(3):376. doi: 10.3390/cancers18030376 (PMC12896816; doi:10.3390/cancers18030376)
Supplement: Supplementary file 1 [file cancers-18-00376-s001.zip › Supplemental Tables S1-S4.pdf]

**Supplemental Table S1:** Human breast tissue donor information				
**BMI**	**Gender**	**Race**	**Age**	**Experiment/Figure(s) Reference**
31.1	F	Black	52	RNA Sequencing; Figure 1, Table 2, Figure 2A; Figure 3, Figure 5
35.9	F	Black	29	
34.9	F	Black	31	
24.9	F	Caucasian	20	
35.9	F	Black	29	Western Blot; Figure 2D
34.9	F	Black	31	
26.1	F	Black	33	
30.6	F	Black	27	Histology; Figure 6
31.7	F	Black	52	
37.8	F	Black	50	
42.8	F	Black	47	
31.1	F	Black	52	
35.9	F	Black	29	
34.9	F	Black	31	
24.9	F	Caucasian	20	
33.1	F	Black	61	Time-lapse Imaging; Figure 4C and 4D, Supplemental Figure 4
33.3	F	Black	34	
30.6	F	Black	27	

**Supplemental Table S2**: Transcriptomic significant pathways in MDA-MB-231-ERK5-ko cells cultured all models			
**Upregulated**		**Downregulated**	
**Pathway**	**P-value**	**Pathway**	**P-value**
Apical Surface Proteins	1.72E-2	Estrogen Response Late	3.68E-2
----	--	KRAS Signaling Late	3.68E-2

**Supplemental Table S3:** Transcriptomic significant pathways in MDA-MB-231-ERK5-ko cells cultured 2D and BA-MaPS			
**Upregulated**		**Downregulated**	
**Pathway**	**P-value**	**Pathway**	**P-value**
Apical Surface	2.03E-03	Estrogen Respones Late	6.04E-03
Estrogen Response Early	3.33E-03	KRAS Signaling Up	6.04E-03
Myogenesis	3.33E-03	Wnt-beta Catenin Signaling	8.89E-03
Complement	1.18E-02	Glycolysis	1.69E-02
Estrogen Response Late	3.02E-02	Notch Signaling	2.40E-02
----		Estrogen Response Early	4.29E-02

**Supplemental Table S4:** NFκB transcript and protein names and function			
**Transcript**	**Protein**	**Function**	**References**
NFKB1	p105/p50<br>P50/p65 (RelA)	NFκB1 precursor is p105 and cleaves to NFκB1 active form, p50. p50 cleaves to p65 (RelA) to create a more powerful activation response. This the most common and active form of NFκB [p50/p65 (RelA)]	[37, 38]
NFKB2	P100/p52	NFκB2 precursor is p100 (also can act as an inhibitor of NFκB2) and cleaves to NFκB2 active form, p52	
NFKBIA	IκBα	Inhibitor of p50/p65 (RelA) NFκB dimer, can also inhibit NFκB2	
NFKBIE	IκBε	Inhibitor of p50/p65 (RelA) and p50/c-Rel dimers	
NFKBIZ	IκBζ	Inhibitor of p50/p65 (RelA)	
